# Supplementary material for: Investigations into the biosynthesis of stieleriacines and related N-acyl tyrosines by comparative genomics, knock-out studies and total synthesis of epi-stieleriacine C
Source: Commun Chem. 2025 Sep 29;8:283. doi: 10.1038/s42004-025-01654-4 (PMC12479983; doi:10.1038/s42004-025-01654-4)
Supplement: Supplementary file 2 — Supplementary Information [file 42004_2025_1654_MOESM2_ESM.pdf]

Supplementary Table 1. Strain information and list of analyzed enzymes.

Species	*Massilia kyonggiensis*	*Massilia namuconiensis*	*Massilia umbonata*	*Stieleria maiorica*	*Stieleria neptunia*
Strain	TSA1 = JCM 19189	333-1-0411 = CGMCC 1.11014	LP01 = DSM 26121	Mal15	Enr13
NCBI acc. No.	GCF_024756235.1	GCF_900116645.1	GCF_005280315.1	GCA_008035925.1	GCA_007754155.1
Putative protein function					
N-acyl amino acid synthase	NX783_RS15985	BM436_RS07845<br>BM436_RS05415<br>BM436_RS05420	FCL38_RS04550<br>FCL38_RS21530<br>FCL38_RS21690	Mal15_37340<br>Mal15_37370<br>Mal15_37380<br>Mal15_37430	Enr13x_30590<br>Enr13x_31280<br>Enr13x_41680
ThiF-like adenylyltransferase	NX783_RS15990	--	--	Mal15_37260	Enr13x_31220
Nitroreductase-like protein	NX783_RS15995	--	--	Mal15_37310	Enr13x_31230
Tyrosine C3-methyltransferase	nd	nd	nd	Mal15_37250	--
Tyrosine O-methyltransferase	nd	nd	nd	--	Enr13x_31210

Supplementary Note 1

NMR spectroscopy: NMR measurements were performed on a Bruker AVANCE II 300 MHz and a Bruker AVANCE 500 MHz spectrometer. The chemical shifts are reported in parts per million (ppm) relative to the solvent residual peak of DMSO-d6 (^{1}H: 2.50 ppm, quintet; ^{13}C: 39.52 ppm, septet), $CDCl_3$ (^{1}H: 7.26 ppm, singlet; ^{13}C: 77.16 ppm, triplet) and CD_3OD (^{1}H: 3.31 ppm, quintet; ^{13}C: 49.00 ppm, septet).

High Resolution Mass Spectrometry: LC-ESI-HRMS measurements were carried out on an Accela UPLC system (Thermo Scientific) coupled with a Kinetex C18 column (150 x 2.1 mm, particle size 2.6 µm) or a Kinetex C8 column (50 x 2.1 mm, particle size 1.7 µm) combined with a Q-Exactive mass spectrometer (Thermo Scientific) equipped with an electrospray ion (ESI) source.

Column chromatography: Flash chromatography was performed on a Biotage Isolera Prime. Normal phase purifications were run on Biotage cartridges packed with Normasil 60 silica gel (particle size 40 – 63 µm).

TLC: Thin layer chromatography was performed using 0.25 mm Macherey-Nagel silica plates with fluorescent indicator UV254, using short-wave UV light as the visualizing agent, $KMnO_4$ and heat as developing agents.

IR Spectroscopy: IR spectra were recorded on an FT/IR-4100 ATR spectrometer (JASCO).

Polarimetry: Optical rotations were recorded on a P-1020 polarimeter (JASCO) at 589 nm using a 50 mm cell and the solvent and concentration (g/100 mL) indicated.

Chemicals: All reagents and solvents for synthesis were purchased from ABCR, Acros Organics, Alfa Aesar, Carbolution Chemicals, Carl Roth, Fluorochem, Sigma Aldrich, TCI, Th. Geyer and used without further purification. All anhydrous solvents were purchased from Acros Organics. Unless otherwise stated all reactions of air/water sensitive substances were carried out using standard Schlenk techniques under a positive pressure of argon.

Supplementary Note 2

Extraction and UHPLC-ESI-HRMS analysis: After cultivation, supernatant and biomass were separated by centrifugation. The cell pellet was extracted for 1 h in 10 mL MeOH, while the culture supernatant was extracted three times with 100 mL EtOAc. Organic fractions were combined and dried in vacuo. UHPLC-HESI-HRMS measurements were carried out on a Vanquish Flex UHPLC system (Thermo Scientific) combined with an Orbitrap Exploris 120 mass spectrometer (Thermo Scientific) equipped with a heated electrospray ionization (HESI) source. Metabolites were separated using reverse phase liquid chromatography at 40 °C using a Kinetex C18 column (50 x 2.1 mm, particle size 1.7 µm, 100 Å, Phenomenex) preceded by a C18 SecurityGuardTM ULTRA guard cartridge (2.1mm, Phenomenex). Mobile phases consisted of H_2O + 0.1 % formic acid (A) and ACN + 0.1 % formic acid (B). 5 µL sample concentrated at 200 µg/mL were injected into a gradient as follows: 0-1 min, 5 % B; 1-10 min, 97 % B; 10-12 min, 97 % B; 12-13 min, 5 % B; 13-15 min, 5 % B at a constant flow rate of 0.3 mL/min. Data dependent acquisition of MS2 spectra was performed in positive mode. HESI parameters were set to 50 AU sheath gas flow, 13 AU auxiliary gas flow, 1 AU sweep gas flow, 3.4 kV (+) spray voltage, 300 °C vaporizer temperature and 320 °C ion transfer tube temperature. MS1 full scan parameters were set to data type-centroid, *m/z* 150-1500 scan range, resolving power 60,000 at *m/z* 200, 1 micro-scan, 100 ms max. injection time, 1E6 automated gain control, 70 % RF lens, dynamic exclusion filter-auto and isotope exclusion filter-assigned. Up to 4 MS2 spectra per MS1 survey scan were recorded with the following parameters, data type-centroid, scan range-auto, resolving power 30,000 at *m/z* 200, 1 micro-scan, 100 ms max. Injection time, 1E5 automated gain control, 1.2 *m/z* isolation window, collision energy type- normalized with a stepwise increase from 20 to 30 to 40 %.

Molecular network analysis: A molecular network was created using the online workflow (https://ccms-ucsd.github.io/GNPSDocumentation/) on the GNPS website (http://gnps.ucsd.edu). The data was filtered by removing all MS/MS fragment ions within +/- 17 Da of the precursor m/z. MS/MS spectra were window filtered by choosing only the top 6 fragment ions in the +/- 50 Da window throughout the spectrum. The precursor ion mass tolerance was set to 0.02 Da and a MS/MS fragment ion tolerance of 0.02 Da. A network was then created where edges were filtered to have a cosine score above 0.8 and more than 6 matched peaks. Further, edges between two nodes were kept in the network if and only if each of the nodes appeared in each other's respective top 10 most similar nodes. Finally, the maximum size of a molecular family was set to 100, and the lowest scoring edges were removed from molecular families until the molecular family size was below this threshold. The spectra in the network were then searched against GNPS' spectral libraries. The library spectra were filtered in the same manner as the input data. All matches kept between network spectra and library spectra were required to have a score above 0.7 and at least 6 matched peaks (DOI:10.1038/nbt.3597). Spectral networks were visualized using Cytoscape 3.10.2 (DOI: 10.1101/gr.1239303).

Data has been deposited on the MassIVE server (MSV000098273; Doi:10.25345/C5F766K86).
https://massive.ucsd.edu/ProteoSAFe/dataset.jsp?task=4e59e6003a4344afad431f7b9b36f050)

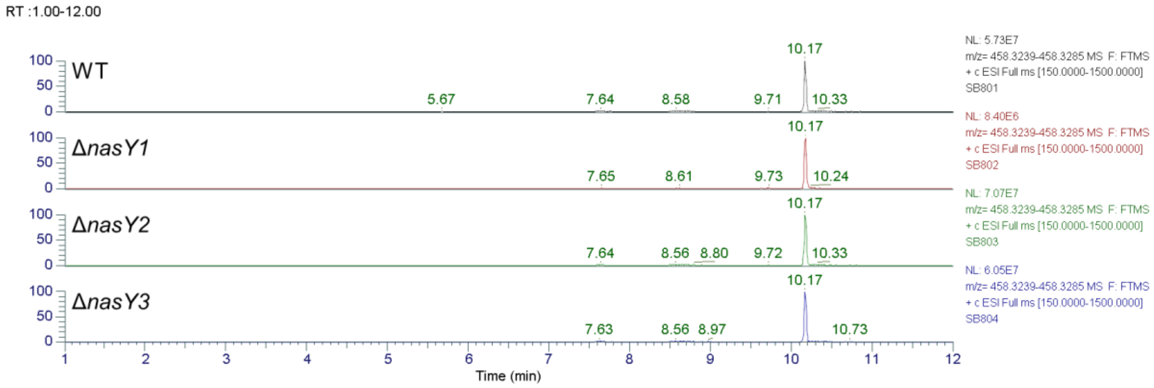

Supplementary Figure 1. Representative extracted ion chromatogram (EIC) targeting the molecular ion feature of stieleriacine D (*m/z* 458.326 $[M+H]^+$). Targeted analysis of extracts obtained from *S. neptunia* Enr13 (black), *S. neptunia* Δ*nasY1* (red), *S. neptunia* Δ*nasY2* (green) and *S. neptunia* Δ*nasY3* (blue)

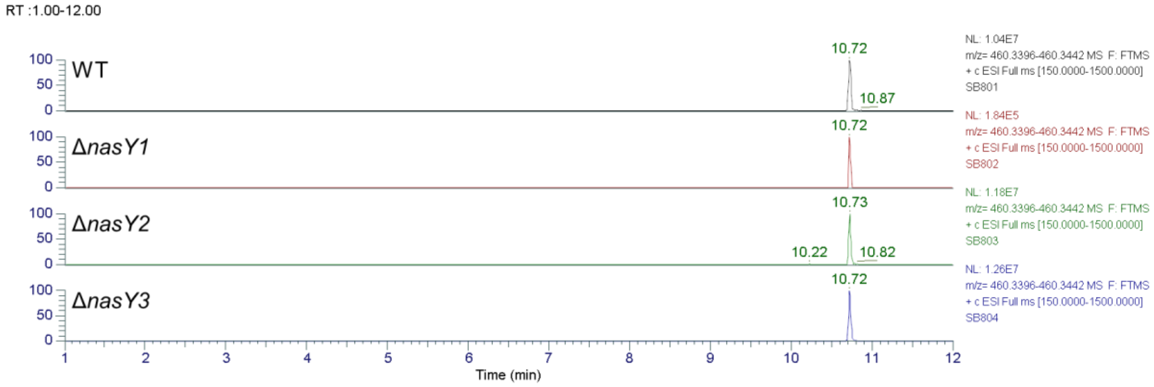

Supplementary Figure 2. Representative extracted ion chromatogram (EIC) analysis targeting stieleriacine E (*m/z* 460.342 $[M+H]^+$). Targeted analysis of extracts obtained from *S. neptunia* Enr13 (black), *S. neptunia* Δ*nasY1* (red), *S. neptunia* Δ*nasY2* (green) and *S. neptunia* Δ*nasY3* (blue).

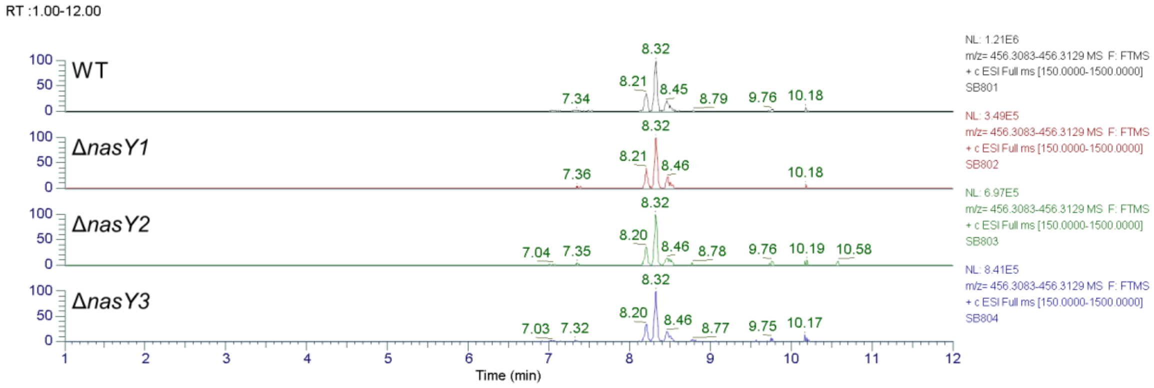

Supplementary Figure 3. Representative extracted ion chromatogram (EIC) analysis targeting stieleriacine F (*m/z* 456.311 $[M+H]^+$). Targeted analysis of extracts obtained from *S. neptunia* Enr13 (black), *S. neptunia* Δ*nasY1* (red), *S. neptunia* Δ*nasY2* (green) and *S. neptunia* Δ*nasY3* (blue) extracts

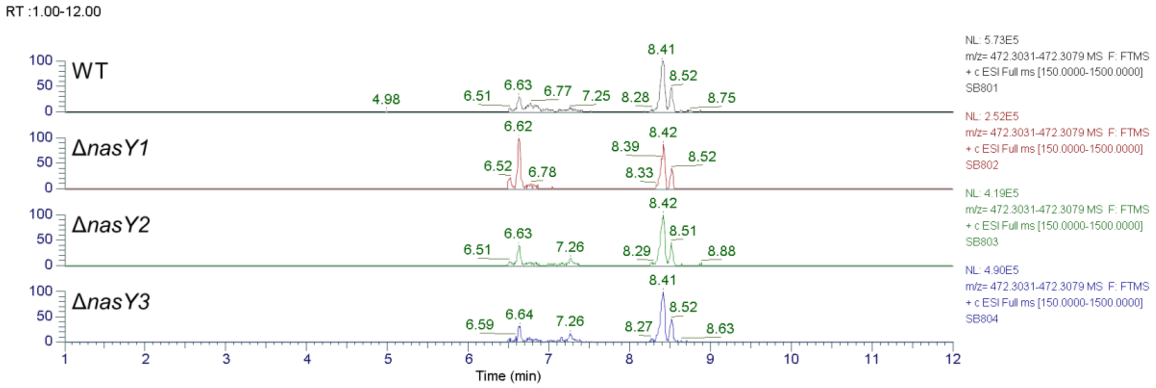

Supplementary Figure 4. Representative extracted ion chromatogram (EIC) analysis targeting stieleriacine G (*m/z* 472.306 [M+H]$^+$). Targeted analysis of extracts obtained from *S. neptunia* Enr13 (black), *S. neptunia* Δ*nasY1* (red), *S. neptunia* Δ*nasY2* (green) and *S. neptunia* Δ*nasY3* (blue) extracts

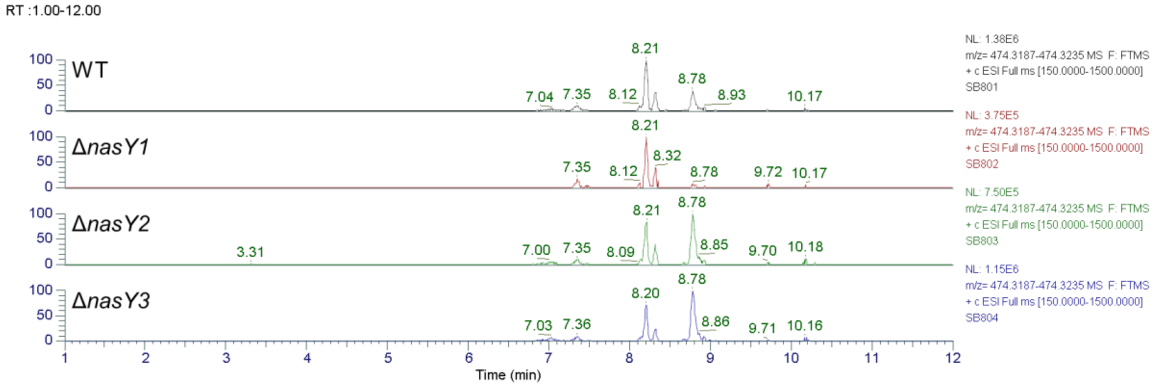

Supplementary Figure 5. Representative extracted ion chromatogram (EIC) analysis targeting stieleriacine H (*m/z* 474.321 [M+H]$^+$). Targeted analysis of extracts obtained from *S. neptunia* Enr13 (black), *S. neptunia* Δ*nasY1* (red), *S. neptunia* Δ*nasY2* (green) and *S. neptunia* Δ*nasY3* (blue).

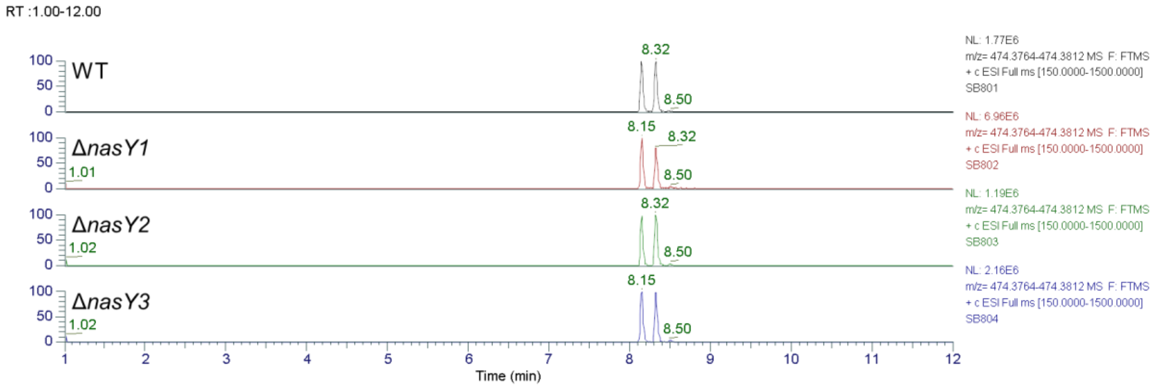

Supplementary Figure 6. Representative extracted ion chromatogram (EIC) analysis targeting *m/z* 474.379 [M+H]$^+$. Targeted analysis of extracts obtained from *S. neptunia* Enr13 (black), *S. neptunia* Δ*nasY1* (red), *S. neptunia* Δ*nasY2* (green) and *S. neptunia* Δ*nasY3* (blue).

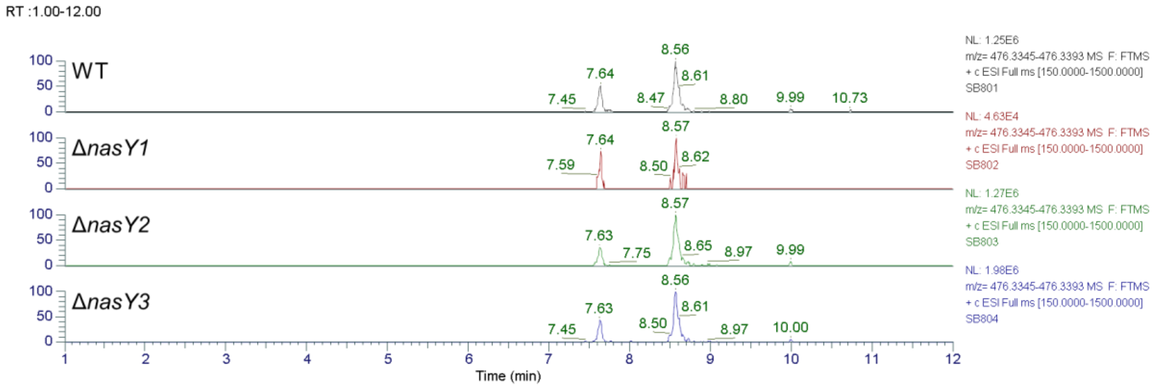

Supplementary Figure 7. Representative extracted ion chromatogram (EIC) analysis targeting stieleriacine I (*m/z* 476.337 $[M+H]^+$). Targeted analysis of extracts obtained from *S. neptunia* Enr13 (black), *S. neptunia* Δ*nasY1* (red), *S. neptunia* Δ*nasY2* (green) and *S. neptunia* Δ*nasY3* (blue) extracts

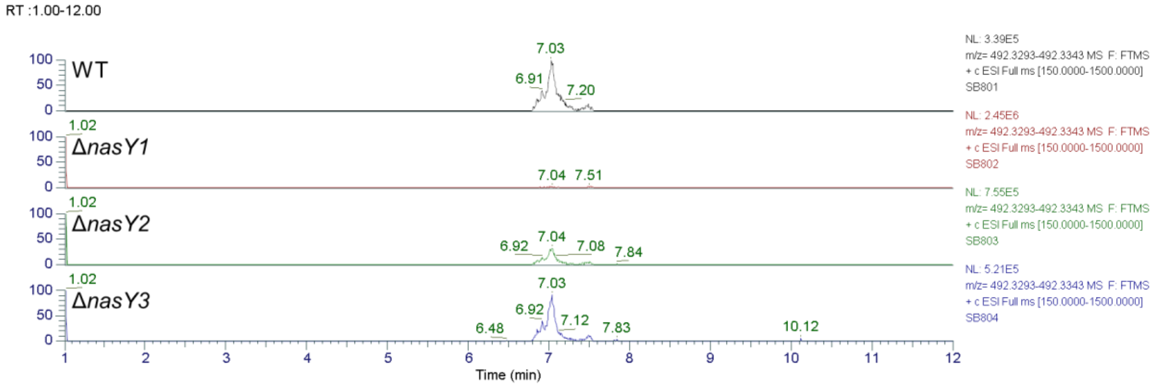

Supplementary Figure 8. Representative extracted ion chromatogram (EIC) analysis targeting *m/z* 492.332 $[M+H]^+$. Targeted analysis of extracts obtained from *S. neptunia* Enr13 (black), *S. neptunia* Δ*nasY1* (red), *S. neptunia* Δ*nasY2* (green) and *S. neptunia* Δ*nasY3* (blue) extracts EIC signal intensity in Δ*nasY1* mutant is elevated due trace amounts of non-related mass feature of the same mass in injection peak.

Supplementary Note 3 - Synthesis of Aminolipids

General synthetic procedures

GP1. General procedure for amide coupling with saturated fatty acid[1]

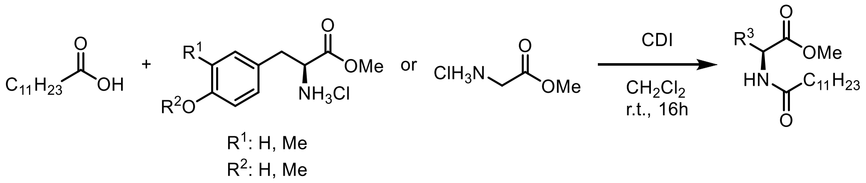

To a solution of lauric acid (1.0 equiv.) in CH_2Cl_2 (0.2 M) was added CDI (1.1 equiv.) and the reaction mixture was stirred at r.t. for 30 min. After addition of amino acid hydrochloride (1.1 equiv.) the reaction mixture was stirred for 16 h. Then diluted with CH_2Cl_2, quenched by the addition of sat. NH_4Cl solution and acidified to pH 2 with 1 M HCl. The organic layer was separated and the aqueous layer was extracted with CH_2Cl_2. The combined organic layers were washed with brine, dried over $MgSO_4$, filtrated and concentrated *in vacuo.* The residue was purified by column chromatography (0 to 50% EtOAc in cyclohexane) to yield the amide as white solid.

GP2. General procedure for amide coupling with unsaturated fatty acid[2]

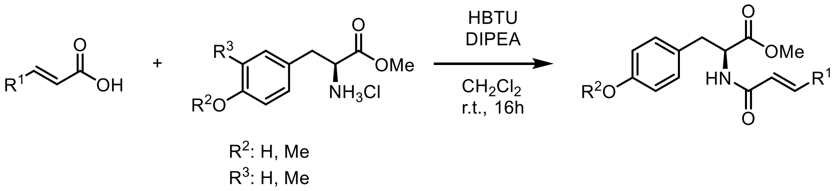

To a solution of unsaturated fatty acid (1.0 equiv.) in CH_2Cl_2 (0.1 M) were added DIPEA (2.5 equiv.) followed by HBTU (1.2 equiv.) and the reaction mixture was stirred at r.t. for 5 min. After the addition of tyrosine derivative (1.2 equiv.) the reaction mixture was stirred for 16 h, quenched by the addition of 10% aqueous citric acid solution and extracted with CH_2Cl_2. The combined organic layers were washed with sat. $NaHCO_3$ solution and brine, dried over $MgSO_4$, filtrated and evaporated *in vacuo*. The residue was purified by column chromatography (0 to 50% EtOAc in cyclohexane) to yield the amide as white solid.

GP3. General procedure for ester hydrolysis[3]

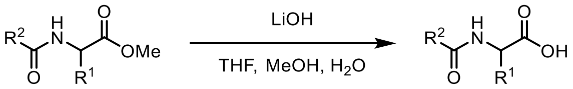

A modified procedure was used: To a solution of methyl ester (1.0 equiv.) in THF, MeOH and water (2:2:1, 0.02-0.08 M) was added lithium hydroxide (25 equiv.) and the reaction mixture was stirred at r.t. for 16 h. The reaction mixture was acidified to pH 2 with 1 M HCl and sodium chloride was added until saturation, followed by cyclohexane. The organic phase was separated and the aqueous layer was extracted with cyclohexane. The combined organic layers were washed with brine, dried over $MgSO_4$, filtrated and concentrated *in vacuo* to yield the amide as white solid.

Synthesis of methyl ester derivatives

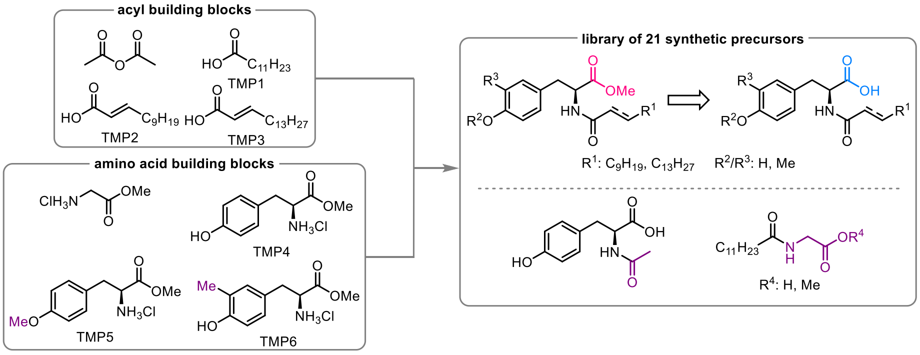

Supplementary Figure 9. General reaction scheme of precursors.

Methyl dodecanoyl-*L*-tyrosinate (18)

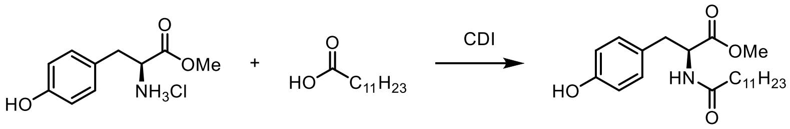

Following GP1: To a solution of lauric acid (500 mg, 2.50 mmol, 1 equiv.) in CH_2Cl_2 (13 mL) was added CDI (445 mg, 2.75 mmol, 1.1 equiv.) followed by H-Tyr-OMe HCl (637 mg, 2.75 mmol, 1.1 equiv.) to yield the amide (566 mg, 60%) as white solid.

^{1}H-NMR (300 MHz, $CDCl_3$) δ = 6.93 (d, *J* = 8.3 Hz, 2H), 6.73 (d, *J* = 8.4 Hz, 2H), 5.99 (d, *J* = 7.9 Hz, 1H), 4.88 (dt, *J* = 8.0, 5.8 Hz, 1H), 3.73 (s, 3H), 3.14 – 2.92 (m, 2H), 2.17 (t, *J* = 7.6 Hz, 2H), 1.65 – 1.51 (m, 2H), 1.25 (s, 16H), 0.87 (t, *J* = 6.6 Hz, 3H) ppm.

^{13}C{^{1}H}-NMR (75 MHz, $CDCl_3$) δ = 173.5, 172.5, 155.6, 130.4, 127.2, 115.7, 53.3, 52.5, 37.4, 36.7, 32.0, 29.7, 29.6, 29.5, 29.4, 29.3, 25.7, 22.8, 14.2 ppm.

HRMS (ESI) calcd for $C_{22}H_{36}NO_4$ $[M+H]^+$ 378.2639, found 378.2644.

The analytical data are in accordance with literature data.[6]

Methyl (*E*)-dodec-2-enoyl-*L*-tyrosinate (19)

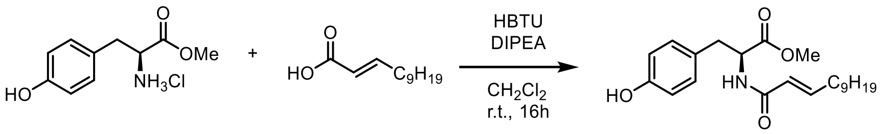

Following GP2: To a solution of (*E*)-dodec-2-enoic acid (50 mg, 0.25 mmol, 1 equiv.), DIPEA (0.11 mL 0.63 mmol, 2.5 equiv.) and HBTU (115 mg, 0.3 mmol, 1.2 equiv.) in CH_2Cl_2 (2.5 mL) was added H-Tyr-OMe HCl (70 mg, 0.3 mmol, 1.2 equiv.) to yield the amide (78.5 mg, 83%) as white solid.

$[\boldsymbol{\alpha}]_{\mathbf{D}}^{\mathbf{20}}$ = 6.3 (c = 0.40 MeOH)

^{1}H-NMR (300 MHz, CDCl$_3$) δ = 6.99 – 6.92 (m, 2H), 6.92 – 6.79 (m, 1H), 6.78 – 6.70 (m, 2H), 5.90 (d, *J* = 7.9 Hz, 1H), 5.76 (dt, *J* = 15.3, 1.5 Hz, 1H), 4.99 – 4.87 (m, 1H), 3.73 (s, 3H), 3.16 – 2.97 (m, 2H), 2.26 – 2.08 (m, 2H), 1.50 – 1.35 (m, 2H), 1.26 (s, 12H), 0.88 (t, *J* = 6.9, 6.4 Hz, 3H) ppm.

^{13}C{^{1}H}-NMR (75 MHz, CDCl$_3$) δ = 172.4, 165.7, 155.1, 146.4, 130.6, 127.8, 123.0, 115.6, 53.4, 52.5, 37.4, 32.3, 32.0, 29.7, 29.6, 29.5, 29.3, 28.3, 22.8, 14.3 ppm.

IR (ATR) $\tilde{\upsilon}$ = 3310 (m), 2914 (s), 2847 (s), 1737 (s), 1671 (m), 1631 (s), 1538 (m), 1513 (s), 1228 (s), 980 (s), 828 (m), 638 (w), 505 (m) cm^{-1}.

HRMS (ESI) calcd for $C_{22}H_{34}NO_4$ $[M+H]^+$ 376.2482, found 376.2487.

Methyl (*E*)-hexadec-2-enoyl-*L*-tyrosinate (20)

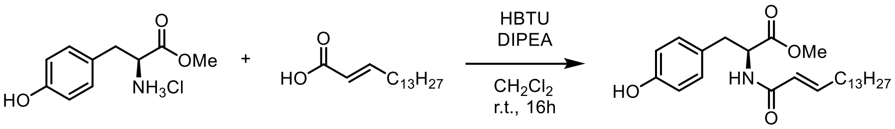

Following GP2: To a solution of (*E*)-hexadec-2-enoic acid (10 mg, 0.04 mmol, 1 equiv.), DIPEA (17 µL 0.10 mmol, 2.5 equiv.) and HBTU (18 mg, 0.05 mmol, 1.2 equiv.) in CH_2Cl_2 (0.4 mL) was added H-Tyr-OMe HCl (11 mg, 0.05 mmol, 1.2 equiv.) to yield the amide (15.4 mg, 91%) as white solid.

$[\boldsymbol{\alpha}]_{\mathbf{D}}^{\mathbf{20}}$ = -3.2 (c = 0.21 MeOH)

^{1}H-NMR (300 MHz, CDCl$_3$) δ = 7.00 – 6.92 (m, 2H), 6.92 – 6.79 (m, 1H), 6.78 – 6.69 (m, 2H), 5.89 (d, *J* = 7.9 Hz, 1H), 5.76 (dt, *J* = 15.3, 1.5 Hz, 1H), 5.00 – 4.86 (m, 1H), 3.73 (s, 3H), 3.17 – 2.99 (m, 2H), 2.22 – 2.11 (m, 2H), 1.42 (q, *J* = 6.9, 6.5 Hz, 2H), 1.26 (s, 20H), 0.88 (t, *J* = 6.6 Hz, 3H) ppm.

^{13}C{^{1}H}-NMR (75 MHz, CDCl$_3$) δ = 172.4, 165.7, 155.1, 146.4, 130.6, 127.8, 123.0, 115.6, 53.4, 52.5, 37.4, 32.3, 32.1, 29.8, 29.8, 29.7, 29.6, 29.5, 29.4, 28.3, 22.8, 14.3 ppm.

IR (ATR) $\tilde{\upsilon}$ = 3309 (m), 2914 (s), 2847 (s), 1738 (s), 1671 (m), 1630 (s), 1540 (m), 1513 (m), 1228 (s), 982 (m), 829 (m), 644 (m) cm^{-1}.

HRMS (ESI) calcd for $C_{26}H_{42}NO_4$ $[M+H]^+$ 432.3108, found 432.3116.

Dodecanoyl-*L*-tyrosine (21)

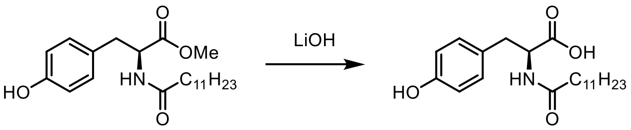

Following GP3: To a solution of methyl ester (200 mg, 0.53 mmol, 1.0 equiv.) in THF, MeOH and water (6.6 mL, 2:2:1) was added lithium hydroxide (317 mg, 13.3 mmol, 25 equiv.) to yield the amide (189 mg, 98%) as white solid.

^{1}H-NMR (500 MHz, DMSO-d6) δ = 12.58 (s, br, 1H), 9.19 (s, br, 1H), 8.03 (d, *J* = 8.1 Hz, 1H), 6.99 (d, *J* = 8.4 Hz, 2H), 6.63 (d, *J* = 8.4 Hz, 2H), 4.37 – 4.26 (m, 1H), 2.90 (dd, *J* = 13.9, 4.8 Hz, 1H), 2.70 (dd, *J* = 13.9, 9.7 Hz, 1H), 2.03 (t, *J* = 7.3 Hz, 2H), 1.38 (p, *J* = 7.5 Hz, 2H), 1.32 – 1.08 (m, 16H), 0.85 (t, *J* = 6.8 Hz, 3H) ppm.

^{13}C{^{1}H}-NMR (126 MHz, DMSO-d6) δ = 173.4, 172.1, 155.9, 130.0, 127.8, 114.9, 114.6, 53.7, 36.0, 35.1, 31.4, 29.1, 29.1, 29.0, 28.9, 28.8, 28.5, 25.2, 22.2, 14.0 ppm.

HRMS (ESI) calcd for $C_{21}H_{34}NO_4$ $[M+H]^+$ 364.2482, found 364.2489.

The analytical data are in accordance with literature data.[7]

(*E*)-Dodec-2-enoyl-*L*-tyrosine (22)

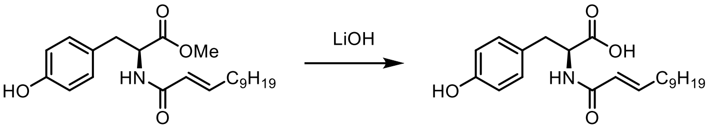

Following GP3: To a solution of methyl ester (25.0 mg, 0.067 mmol, 1.0 equiv.) in THF, MeOH and water (0.83 mL, 2:2:1) was added lithium hydroxide (40.0 mg, 1.68 mmol, 25 equiv.) to yield the amide (23.8 mg, 99%) as white solid.

$[\alpha]_D^{20}$ = 28.7 (c = 0.25 MeOH)

^{1}H-NMR (500 MHz, DMSO-d6) δ = 12.20 (s, br, 1H), 9.22 (s, br, 1H), 8.12 (d, *J* = 8.1 Hz, 1H), 7.00 (d, *J* = 8.1 Hz, 2H), 6.63 (d, *J* = 8.1 Hz, 2H), 6.55 (dt, *J* = 14.5, 6.9 Hz, 1H), 5.93 (d, *J* = 15.3 Hz, 1H), 4.42 – 4.30 (m, 1H), 2.94 (dd, *J* = 13.9, 4.7 Hz, 1H), 2.73 (dd, *J* = 13.9, 9.4 Hz, 1H), 2.15 – 2.02 (m, 2H), 1.44 – 1.31 (m, 2H), 1.24 (s, 12H), 0.85 (t, *J* = 6.7 Hz, 3H) ppm.

^{13}C{^{1}H}-NMR (126 MHz, DMSO-d6) δ = 173.6, 164.8, 155.8, 143.1, 130.0, 127.9, 124.1, 114.9, 54.1, 36.1, 31.3, 31.2, 29.0, 28.9, 28.8, 28.7, 27.9, 22.1, 14.0 ppm.

IR (ATR) $\tilde{\nu}$ = 3296 (m), 3231 (m), 2916 (s), 2848 (s), 1704 (s), 1634 (s), 1549 (s), 1514 (s), 1454 (m), 1227 (s), 1105 (m), 823 (m), 666 (s), 538 (s) cm^{-1}.

HRMS (ESI) calcd for $C_{21}H_{32}NO_4$ $[M+H]^+$ 362.2326, found 362.2330.

(*E*)-Hexadec-2-enoyl-*L*-tyrosine (23)

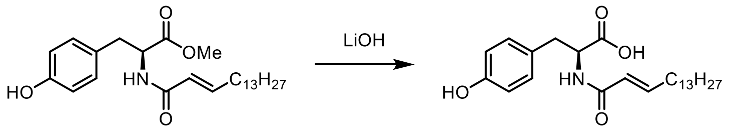

Following GP3: To a solution of methyl ester (5.0 mg, 0.012 mmol, 1.0 equiv.) in THF, MeOH and water (0.5 mL, 2:2:1) was added lithium hydroxide (7.0 mg, 0.29 mmol, 25 equiv.) to yield the amide (4.8 mg, 99%) as white solid.

$[\alpha]_D^{20}$ = 25.3 (c = 0.14 MeOH)

^{1}H-NMR (500 MHz, CD_3OD) δ = 7.03 (d, *J* = 8.5 Hz, 2H), 6.74 (dt, *J* = 15.4, 7.0 Hz, 1H), 6.68 (d, *J* = 8.5 Hz, 2H), 5.95 (d, *J* = 15.4 Hz, 1H), 4.62 (dd, *J* = 8.5, 5.2 Hz, 1H), 3.11 (dd, *J* = 14.0, 5.2 Hz, 1H), 2.89 (dd, *J* = 14.0, 8.6 Hz, 1H), 2.22 – 2.14 (m, 2H), 1.49 – 1.41 (m, 2H), 1.36 – 1.26 (m, 20H), 0.90 (t, *J* = 6.9 Hz, 3H) ppm.

^{13}C{^{1}H}-NMR (126 MHz, CD_3OD) δ = 168.5, 157.3, 146.4, 131.2, 129.2, 124.3, 116.1, 55.7, 37.8, 33.1, 33.0, 30.8, 30.7, 30.6, 30.5, 30.3, 29.4, 23.8, 14.5 ppm.

HRMS (ESI) calcd for $C_{25}H_{40}NO_4$ $[M+H]^+$ 418.2952, found 418.2957.

Methyl (*S*)-2-dodecanamido-3-(4-methoxyphenyl)propanoate (24)

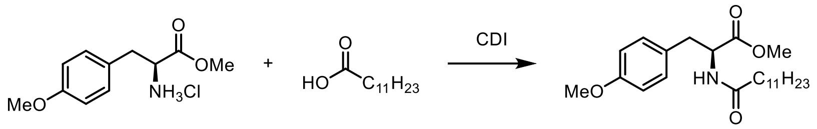

Following GP1: Lauric acid (371 mg, 1.85 mmol, 1.0 equiv.), CDI (331 mg, 2.04 mmol, 1.1 equiv.) and H-Tyr(Me)-OMe HCl (500 mg, 2.04 mmol, 1.1 equiv.) in CH_2Cl_2 (9.0 mL) were used to yield the amide (594 mg, 82%) as white solid.

$[\alpha]_D^{20}$ = 6.6 (c = 2.48 MeOH)

^{1}H-NMR (300 MHz, $CDCl_3$) δ = 7.00 (d, *J* = 8.6 Hz, 2H), 6.81 (d, *J* = 8.7 Hz, 2H), 5.86 (d, *J* = 7.8 Hz, 1H), 4.86 (dt, *J* = 7.9, 5.7 Hz, 1H), 3.78 (s, 3H), 3.72 (s, 3H), 3.16 – 2.96 (m, 2H), 2.16 (t, *J* = 8.0, 7.1 Hz, 2H), 1.68 – 1.50 (m, 2H), 1.25 (s, 16H), 0.87 (t, *J* = 7.1, 6.3 Hz, 3H) ppm.

^{13}C{^{1}H}-NMR (75 MHz, $CDCl_3$) δ = 172.8, 172.4, 158.8, 130.4, 127.9, 114.1, 55.3, 53.2, 52.4, 37.2, 36.7, 32.0, 29.8, 29.6, 29.5, 29.4, 25.7, 22.8, 14.2 ppm.

IR (ATR) $\tilde{\upsilon}$ = 3337 (m), 2914 (s), 2848 (s), 1749 (s), 1647 (s), 1515 (s), 1431 (w), 1244 (s), 1164 (s), 1032 (m), 829 (m), 652 (w), 556 (m) cm^{-1}.

HRMS (ESI) calcd for $C_{23}H_{38}NO_4$ $[M+H]^+$ 392.2795, found 392.2801.

Methyl (*S,E*)-2-(dodec-2-enamido)-3-(4-methoxyphenyl)propanoate (25)

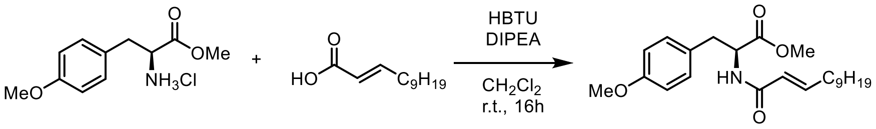

Following GP2: To a solution of (*E*)-dodec-2-enoic acid (50 mg, 0.25 mmol, 1 equiv.), DIPEA (0.11 mL 0.63 mmol, 2.5 equiv.) and HBTU (115 mg, 0.3 mmol, 1.2 equiv.) in CH_2Cl_2 (2.5 mL) was added H-Tyr(Me)-OMe HCl (74 mg, 0.3 mmol, 1.2 equiv.) to yield the amide (84.1 mg, 89%) as white solid.

$[\alpha]_D^{20}$ = 1.0 (c = 0.43 MeOH)

^{1}H-NMR (300 MHz, $CDCl_3$) δ = 7.04 – 6.97 (m, 2H), 6.90 – 6.78 (m, 3H), 5.87 (d, *J* = 7.7 Hz, 1H), 5.76 (dt, *J* = 15.3, 1.5 Hz, 1H), 4.98 – 4.87 (m, 1H), 3.78 (s, 3H), 3.73 (s, 3H), 3.10 (dd, *J* = 5.6, 2.0 Hz, 2H), 2.24 – 2.11 (m, 2H), 1.51 – 1.37 (m, 2H), 1.34 – 1.21 (m, 12H), 0.88 (t, *J* = 7.0, 6.5 Hz, 3H) ppm.

^{13}C{^{1}H}-NMR (75 MHz, $CDCl_3$) δ = 172.3, 165.6, 158.8, 146.1, 130.4, 127.9, 123.1, 114.1, 55.3, 53.3, 52.4, 37.2, 32.2, 32.0, 29.7, 29.6, 29.4, 29.3, 28.3, 22.8, 14.2 ppm.

IR (ATR) $\tilde{\upsilon}$ = 3317 (m), 2914 (s), 2847 (s), 1737 (s), 1671 (m), 1631 (s), 1538 (m), 1514 (s), 1248 (s), 1180 (s), 1030 (s), 980 (s), 841 (m), 818 (m), 638 (m), 551 (m) cm^{-1}.

HRMS (ESI) calcd for $C_{23}H_{36}NO_4$ $[M+H]^+$ 390.2639, found 390.2643.

Methyl (*S,E*)-2-(hexadec-2-enamido)-3-(4-methoxyphenyl)propanoate (26)

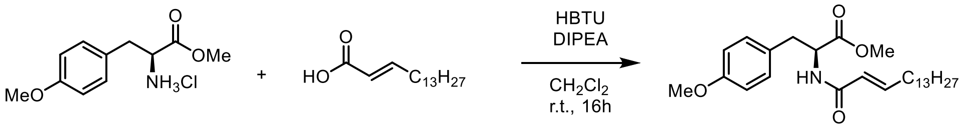

Following GP2: To a solution of (*E*)-hexadec-2-enoic acid (10 mg, 0.04 mmol, 1 equiv.), DIPEA (17 µL 0.10 mmol, 2.5 equiv.) and HBTU (18 mg, 0.05 mmol, 1.2 equiv.) in CH_2Cl_2 (0.4 mL) was added H-Tyr(Me)-OMe HCl (12 mg, 0.05 mmol, 1.2 equiv.) to yield the amide (16.0 mg, 94%) as white solid.

$[\alpha]_D^{20}$ = -0.5 (c = 0.31 MeOH)

^{1}H-NMR (500 MHz, $CDCl_3$) δ = 7.03 – 6.98 (m, 2H), 6.89 – 6.84 (m, 1H), 6.83 – 6.80 (m, 2H), 5.84 (d, *J* = 7.8 Hz, 1H), 5.76 (dd, *J* = 15.3, 1.6 Hz, 1H), 4.92 (dt, *J* = 7.8, 5.5 Hz, 1H), 3.78 (s, 3H), 3.73 (s, 3H), 3.17 – 3.02 (m, 2H), 2.17 (td, *J* = 7.1, 5.6 Hz, 2H), 1.43 (p, *J* = 7.3 Hz, 2H), 1.26 (s, 20H), 0.88 (t, *J* = 6.8 Hz, 3H) ppm.

^{13}C{^{1}H}-NMR (126 MHz, $CDCl_3$) δ = 172.3, 165.5, 158.9, 146.1, 130.5, 127.9, 123.1, 114.1, 55.4, 53.4, 52.4, 37.2, 32.2, 32.1, 29.9, 29.8, 29.8, 29.7, 29.6, 29.5, 29.4, 28.3, 22.8, 14.3 ppm.

IR (ATR) $\tilde{\upsilon}$ = 3308 (m), 2913 (s), 2847 (s), 1739 (s), 1671 (m), 1630 (s), 1541 (m), 1515 (m), 1249 (s), 1031 (m), 841 (m), 505 (s) cm^{-1}.

HRMS (ESI) calcd for $C_{27}H_{44}NO_4$ $[M+H]^+$ 446.3265, found 446.3251.

(*S*)-2-Dodecanamido-3-(4-methoxyphenyl)propanoic acid (27)

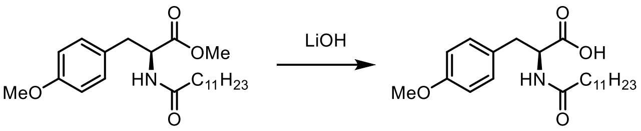

Following GP3: To a solution of methyl ester (300 mg, 0.77 mmol, 1.0 equiv.) in THF, MeOH and water (10 mL, 2:2:1) was added lithium hydroxide (461 mg, 19.3 mmol, 25 equiv.) to yield the amide (280 mg, 97%) as white solid.

^{1}H-NMR (300 MHz, DMSO-d6) δ = 12.49 (s, 1H), 8.01 (d, *J* = 8.2 Hz, 1H), 7.13 (d, *J* = 8.6 Hz, 2H), 6.81 (d, *J* = 8.6 Hz, 2H), 4.42 – 4.30 (m, 1H), 3.70 (s, 3H), 2.97 (dd, *J* = 13.8, 4.8 Hz, 1H), 2.82 – 2.68 (m, 1H), 2.02 (t, *J* = 7.3 Hz, 2H), 1.47 – 1.34 (m, 2H), 1.34 – 1.03 (m, 16H), 0.86 (t, *J* = 6.4 Hz, 3H) ppm.

^{13}C{^{1}H}-NMR (75 MHz, DMSO-d6) δ = 173.3, 172.1, 157.8, 130.0, 129.6, 113.5, 54.9, 53.5, 35.9, 35.1, 31.3, 29.0, 29.0, 28.9, 28.8, 28.7, 28.4, 25.2, 22.1, 13.9 ppm.

HRMS (ESI) calcd for $C_{22}H_{36}NO_4$ $[M+H]^+$ 378.2639, found 378.2644.

(*S,E*)-2-(dodec-2-enamido)-3-(4-methoxyphenyl)propanoic acid (28)

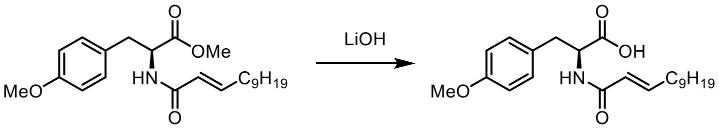

Following GP3: To a solution of methyl ester (25.0 mg, 0.06 mmol, 1.0 equiv.) in THF, MeOH and water (0.75 mL, 2:2:1) was added lithium hydroxide (35.9 mg, 1.5 mmol, 25 equiv.) to yield the amide (24.0 mg, 100%) as white solid.

$[\boldsymbol{\alpha}]_{\mathbf{D}}^{\mathbf{20}}$ = 24.4 (c = 0.28 MeOH)

^{1}H-NMR (500 MHz, DMSO-d6) δ = 12.74 (s, br, 1H), 8.17 (d, *J* = 8.1 Hz, 1H), 7.13 (d, *J* = 8.5 Hz, 2H), 6.82 (d, *J* = 8.6 Hz, 2H), 6.56 (dt, *J* = 15.4, 6.9 Hz, 1H), 5.93 (dd, *J* = 15.4, 1.6 Hz, 1H), 4.45 – 4.33 (m, 1H), 3.70 (s, 3H), 2.99 (dd, *J* = 13.9, 4.8 Hz, 1H), 2.79 (dd, *J* = 13.9, 9.6 Hz, 1H), 2.14 – 2.05 (m, 2H), 1.42 – 1.32 (m, 2H), 1.24 (s, 12H), 0.85 (t, *J* = 6.8 Hz, 3H) ppm.

^{13}C{^{1}H}-NMR (126 MHz, DMSO-d6) δ = 173.3, 164.8, 157.9, 143.1, 130.1, 129.6, 124.0, 113.6, 54.9, 53.9, 36.0, 31.3, 31.2, 29.0, 28.9, 28.7, 28.6, 27.8, 22.1, 14.0 ppm.

IR (ATR) $\tilde{\nu}$ = 3583 (w), 3334 (m), 2916 (s), 2849 (s), 1735 (s), 1665 (m), 1560 (s), 1513 (s), 1443 (m), 1067 (m), 832 (w), 612 (m) cm^{-1}.

HRMS (ESI) calcd for $C_{22}H_{34}NO_4$ $[M+H]^+$ 376.2482, found 376.2488.

(*S*,*E*)-2-(hexadec-2-enamido)-3-(4-methoxyphenyl)propanoic acid (29)

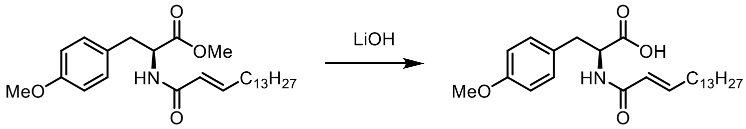

Following GP3: To a solution of methyl ester (10.0 mg, 0.022 mmol, 1.0 equiv.) in THF, MeOH and water (0.5 mL, 2:2:1) was added lithium hydroxide (13.0 mg, 0.56 mmol, 25 equiv.) to yield the amide (9.5 mg, 98%) as white solid.

$[\boldsymbol{\alpha}]_{\mathbf{D}}^{\mathbf{20}}$ = 21.7 (c = 0.18 MeOH)

^{1}H-NMR (500 MHz, CD_3OD) δ = 7.13 (d, *J* = 8.6 Hz, 2H), 6.81 (d, *J* = 8.6 Hz, 2H), 6.73 (dt, *J* = 15.4, 7.0 Hz, 1H), 5.94 (d, *J* = 15.4 Hz, 1H), 4.63 (dd, *J* = 8.6, 5.1 Hz, 1H), 3.74 (s, 3H), 3.15 (dd, *J* = 14.0, 5.1 Hz, 1H), 2.92 (dd, *J* = 14.0, 8.7 Hz, 1H), 2.21 – 2.13 (m, 2H), 1.49 – 1.39 (m, 2H), 1.35 – 1.26 (m, 20H), 0.90 (t, *J* = 6.9 Hz, 3H) ppm.

^{13}C{^{1}H}-NMR (126 MHz, CD_3OD) δ = 168.4, 160.0, 146.4, 131.2, 130.5, 124.3, 114.8, 55.8, 55.6, 37.7, 33.1, 33.0, 30.8, 30.7, 30.5, 30.5, 30.2, 29.4, 23.7, 14.5 ppm.

IR (ATR) $\tilde{\nu}$ = 3208 (m, br), 2918 (m), 2849 (m), 1667 (m), 1513 (m), 1247 (m), 1055 (s) cm^{-1}.

HRMS (ESI) calcd for $C_{26}H_{42}NO_4$ $[M+H]^+$ 432.3108, found 432.3117.

(*S*)-2-acetamido-3-(4-methoxyphenyl)propanoic acid (30)

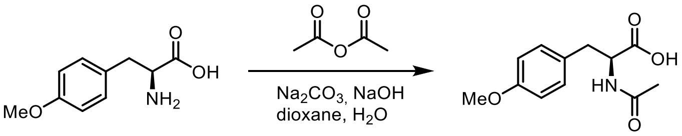

Procedure:[7] A suspension of H-Tyr(Me)-OH (976 mg, 5.00 mmol, 1.0 equiv.) and Na_2CO_3 (795 mg, 7.50 mmol, 1.5 equiv.) in dioxane (5 mL) and water (10 mL) was cooled to 0 °C. Then 1 mL of an acetic anhydride (0.52 mL, 5.5 mmol, 1.1 equiv.) solution in dioxane (5 mL) and 2 mL of a solution of NaOH (260 mg, 6.5 mmol, 1.3 equiv.) in water (10 mL) were added. The rest of the solutions were added in four equivalent portions every 10 min. The cooling bath was removed and the reaction mixture was stirred for 2 h, acidified to pH 2 with 1 M aqueous HCl solution and extracted with EtOAc. The combined organic layers were washed with brine, dried over Na_2SO_4, filtrated and evaporated *in vacuo*. The oily residue was crystallized at 4 °C for 20 h from a minimum volume of EtOAc and hexane. The solid was collected, washed with hexane and dried to yield the amide (771 mg, 65%) as white solid.

^{1}H-NMR (300 MHz, DMSO-d6) δ = 12.61 (s, 1H), 8.12 (d, *J* = 8.1 Hz, 1H), 7.13 (d, *J* = 8.6 Hz, 2H), 6.83 (d, *J* = 8.7 Hz, 1H), 4.40 – 4.27 (m, 1H), 3.71 (s, 3H), 2.96 (dd, *J* = 13.8, 5.0 Hz, 1H), 2.77 (dd, *J* = 9.3, 4.6 Hz, 1H), 1.78 (s, 3H) ppm.

^{13}C{^{1}H}-NMR (75 MHz, DMSO-d6) δ = 173.2, 169.1, 157.8, 130.0, 129.5, 113.6, 54.9, 53.7, 36.0, 22.3 ppm.

HRMS (ESI) calcd for $C_{12}H_{16}NO_4$ $[M+H]^+$ 238.1074, found 238.1079.

The analytical data are in accordance with literature data.[7]

Methyl (*S*)-2-amino-3-(4-hydroxy-3-methylphenyl)propanoate hydrochloride

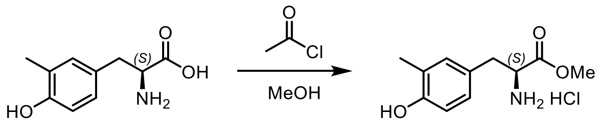

Following a modified procedure:[4] Acetyl chloride (0.26 mL, 3.59 mmol, 7.0 equiv.) was added to MeOH (2 mL), followed by H-L-Tyr(3-Me)-OH (100 mg, 0.51 mmol 1.0 equiv.). The reaction mixture was stirred under reflux for 16 h, cooled to r.t. and evaporated to yield the ester (135.2 mg, 107%) as brown solid, which was used without further purification.

^{1}H-NMR (500 MHz, CD_3OD) δ = 6.96 (d, *J* = 2.2 Hz, 1H), 6.88 (dd, *J* = 8.2, 2.2 Hz, 1H), 6.74 (d, *J* = 8.1 Hz, 1H), 4.28 – 4.20 (m, 1H), 3.81 (s, 3H), 3.14 (dd, *J* = 14.5, 5.7 Hz, 1H), 3.04 (dd, *J* = 14.5, 7.4 Hz, 1H), 2.18 (s, 3H) ppm.

^{13}C{^{1}H}-NMR (126 MHz, CD_3OD) δ = 170.6, 156.4, 132.7, 128.7, 126.4, 125.4, 116.1, 55.4, 53.5, 36.7, 16.2 ppm.

HRMS (ESI): calcd for $C_{11}H_{16}NO_3$ $[M+H]^+$ 210.1125, found 210.1132.

Methyl (*S*)-2-dodecanamido-3-(4-hydroxy-3-methylphenyl)propanoate (31)

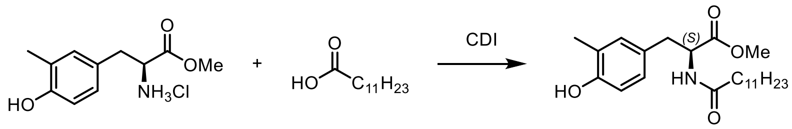

Following GP1: To a solution of lauric acid (36.1 mg, 0.18 mmol, 1.0 equiv.) in CH_2Cl_2 (1.0 mL) was added CDI (32.4 mg, 0.20 mmol, 1.1 equiv.) followed by H-Tyr(3-Me)-OMe HCl (50 mg, 0.20 mmol, 1.1 equiv.) to yield the amide (54.6 mg, 77%) as brownish solid.

$[\alpha]_D^{20}$ = 18.3 (c = 0.14 MeOH)

^{1}H-NMR (500 MHz, $CDCl_3$) δ = 6.81 (d, *J* = 2.1 Hz, 1H), 6.75 (dd, *J* = 8.1, 2.2 Hz, 1H), 6.64 (d, *J* = 8.1 Hz, 1H), 6.01 (d, *J* = 7.9 Hz, 1H), 5.79 (s,br, 1H), 4.90 – 4.81 (m, 1H), 3.74 (s, 3H), 3.03 (dd, *J* = 14.0, 5.7 Hz, 1H), 2.97 (dd, *J* = 14.1, 5.7 Hz, 1H), 2.23 – 2.15 (m, 5H), 1.67 – 1.54 (m, 2H), 1.33 – 1.21 (m, 16H), 0.87 (t, *J* = 6.8 Hz, 3H) ppm.

^{13}C{^{1}H}-NMR (126MHz, $CDCl_3$) δ = 178.6, 173.4, 172.7, 153.5, 132.0, 127.7, 127.3, 124.2, 115.1, 53.2, 52.5, 37.3, 36.7, 34.0, 32.0, 29.8, 29.7, 29.6, 29.5, 29.4, 29.4, 29.2, 25.8, 24.9, 22.8, 16.0, 14.3 ppm.

IR (ATR) $\tilde{\upsilon}$ = 3455 (m), 3303 (m), 2917 (s), 2849 (s), 1727 (s), 1646 (s), 1537 (m), 1434 (w), 1274 (s), 1223 (s), 1114 (s), 814 (m), 675 (w), 596 (m) cm^{-1}.

HRMS (ESI) calcd for $C_{23}H_{38}NO_4$ $[M+H]^+$ 392.2795, found 392.2808.

Methyl (*S*,*E*)-2-(dodec-2-enamido)-3-(4-hydroxy-3-methylphenyl)propanoate (32)

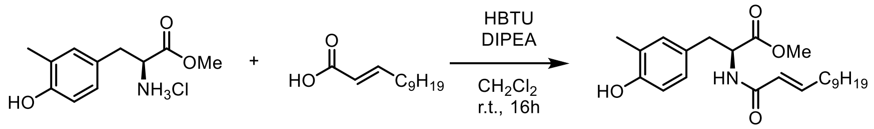

Following GP2: To a solution of (*E*)-dodec-2-enoic acid (43.5 mg, 0.219 mmol, 1.0 equiv.), DIPEA (0.09 mL, 0.548 mmol, 2.5 equiv.) and HBTU (99.7 mg, 0.263 mmol, 1.2 equiv.) in CH_2Cl_2 (2.2 mL) was added H-Tyr(3-Me)-OMe HCl (64.7 mg, 0.263 mmol, 1.2 equiv.) to yield the amide (80.0 mg, 94%) as slightly yellow solid.

$[\alpha]_D^{20}$ = 15.1 (c = 0.12 MeOH)

^{1}H-NMR (500 MHz, $CDCl_3$) δ = 6.89 – 6.81 (m, 2H), 6.80 – 6.73 (m, 1H), 6.65 (dd, *J* = 8.1, 3.2 Hz, 1H), 5.90 (t, *J* = 8.0 Hz, 1H), 5.77 (d, *J* = 15.3 Hz, 1H), 4.95 – 4.87 (m, 1H), 3.73 (s, 3H), 3.10 – 2.99 (m, 2H), 2.21 – 2.15 (m, 5H), 1.48 – 1.39 (m, 2H), 1.31 – 1.20 (m, 12H), 0.88 (t, *J* = 6.8 Hz, 3H) ppm.

^{13}C{^{1}H}-NMR (126MHz, $CDCl_3$) δ = 172.5, 165.7, 153.2, 146.3, 132.1, 127.8, 124.1, 123.0, 115.1, 53.4, 52.5, 37.2, 32.2, 32.0, 29.8, 29.6, 29.6, 29.4, 29.3, 28.3, 22.8, 15.9, 14.3 ppm.

IR (ATR) $\tilde{\upsilon}$ = 3447 (w), 3310 (m), 2919 (s), 2850 (s), 1737 (s), 1708 (s), 1665 (m), 1632 (s), 1531 (s), 1509 (s), 1259 (s), 1210 (s), 1113 (s), 1021 (m), 984 (s), 822 (m), 616 (m), 504 (m) cm^{-1}.

HRMS (ESI) calcd for $C_{23}H_{36}NO_4$ $[M+H]^+$ 390.2639, found 390.2653.

Methyl (*S*,*E*)-2-(hexadec-2-enamido)-3-(4-hydroxy-3-methylphenyl)propanoate (33)

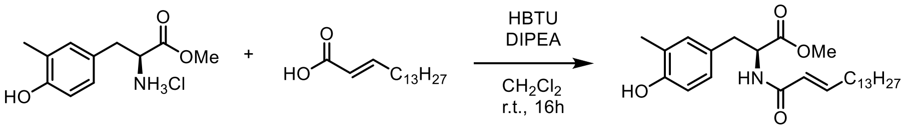

Following GP2: To a solution of (*E*)-hexadec-2-enoic acid (65.1 mg, 0.256 mmol, 1.0 equiv.), DIPEA (0.11 mL, 0.64 mmol, 2.5 equiv.) and HBTU (116.5 mg, 0.31 mmol, 1.2 equiv.) in CH_2Cl_2 (2.6 mL) was added H-Tyr(3-Me)-OMe HCl (0.307 mmol, 1.2 equiv.) to yield the amide (82 mg, 72% over 2 steps) as white solid.

$[\alpha]_D^{20}$ = 7.4 (c = 0.25 MeOH)

^{1}H-NMR (500 MHz, $CDCl_3$) δ = 6.90 – 6.80 (m, 2H), 6.76 (dd, *J* = 8.1, 2.2 Hz, 1H), 6.64 (d, *J* = 8.1 Hz, 1H), 5.95 (d, *J* = 7.9 Hz, 1H), 5.77 (dt, *J* = 15.3, 1.6 Hz, 1H), 4.96 – 4.87 (m, 1H), 3.73 (s, 3H), 3.10 – 2.99 (m, 2H), 2.21 – 2.13 (m, 5H), 1.47 – 1.37 (m, 2H), 1.25 (s, 20H), 0.87 (t, *J* = 6.8 Hz, 3H) ppm.

^{13}C{^{1}H}-NMR (126 MHz, $CDCl_3$) δ = 172.6, 165.8, 153.4, 146.4, 132.0, 127.8, 127.4, 124.2, 122.9, 115.1, 53.4, 52.5, 37.3, 32.2, 32.1, 29.8, 29.8, 29.7, 29.6, 29.5, 29.3, 28.3, 22.8, 16.0, 14.3 ppm.

IR (ATR) $\tilde{\upsilon}$ = 3451 (m), 3315 (m), 2917 (s), 2849 (s), 1737 (s), 1708 (s), 1631 (s), 1531 (s), 1510 (s), 1371 (m), 1263 (s), 1208 (s), 1115 (s), 1208 (s), 1115 (m), 978 (m), 822 (m), 617 (m) cm^{-1}.

HRMS (ESI) calcd for $C_{27}H_{44}NO_4$ $[M+H]^+$ 446.3265, found 446.3265.

Epi-Stieleriacine C - (*S*)-2-Dodecanamido-3-(4-hydroxy-3-methylphenyl)propanoic acid (34)

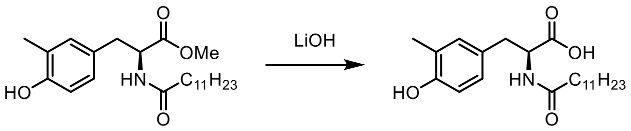

Following modified GP3: To a solution of methyl ester (28.9 mg, 0.07 mmol, 1.0 equiv.) in THF, MeOH and water (0.9 mL, 2:2:1) was added lithium hydroxide (44.2 mg, 1.85 mmol, 25 equiv.). The aqueous phase was extracted with CH_2Cl_2, to yield the amide (6.8 mg, 28%) as solid.

$[\alpha]_D^{20}$ = 28.3 (c = 0.15 MeOH); [isolated **Stieleriacine C** $[\alpha]_D^{21}$ = -28 (c = 1 MeOH)][8]

¹H-NMR (500 MHz, DMSO-d6) δ = 9.08 (s, br, 1H), 7.88 (d, br, *J* = 7.7 Hz, 1H), 6.86 (s, 1H), 6.79 (dd, *J* = 8.2, 2.2 Hz, 1H), 6.62 (d, *J* = 8.1 Hz, 1H), 4.30 – 4.18 (m, 1H), 2.87 (dd, *J* = 13.8, 4.8 Hz, 1H), 2.67 (dd, *J* = 13.8, 9.1 Hz, 1H), 2.04 (s, 3H), 2.03 – 1.99 (m, 2H), 1.43 – 1.34 (m, 2H), 1.21 (m, 14H), 1.13 (m, 2H), 0.85 (t, *J* = 6.7 Hz, 3H) ppm.

¹³C{¹H}-NMR (126 MHz, DMSO-d6) δ = 173.5, 172.1, 153.9, 131.4, 128.0, 127.3, 123.2, 114.3, 54.1, 36.2, 35.3, 31.4, 29.2, 29.1, 29.0, 28.9, 28.8, 28.6, 25.4, 22.2, 16.1, 14.1 ppm.

IR (ATR) $\tilde{\upsilon}$ = 3302 (m), 2918 (s), 2849 (s), 1714 (s), 1645 (s), 1540 (s), 1509 (s), 1261 (s), 1214 (s), 1119 (s), 816 (m), 507 (m) cm^{-1}.

HRMS (ESI) calcd for $C_{22}H_{36}NO_4$ $[M+H]^+$ 378.2639, found 378.2654.

(*S,E*)-2-(Dodec-2-enamido)-3-(4-hydroxy-3-methylphenyl)propanoic acid (35)

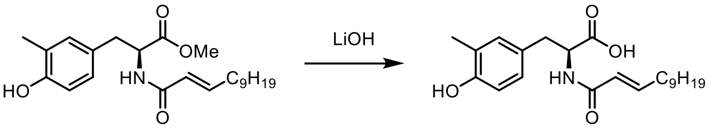

Following modified GP3: To a solution of methyl ester (40.0 mg, 0.103 mmol, 1.0 equiv.) in THF, MeOH and water (1.3 mL, 2:2:1) was added lithium hydroxide (61.7 mg, 2.58 mmol, 25 equiv.). The aqueous phase was extracted with CH_2Cl_2, to yield the amide (12.6 mg, 33%) as solid.

¹H-NMR (500 MHz, CD_3OD) δ = 6.91 (s, 1H), 6.84 (dd, *J* = 8.2, 2.2 Hz, 1H), 6.74 (dt, *J* = 15.3, 7.0 Hz, 1H), 6.64 (d, *J* = 8.2 Hz, 1H), 5.95 (d, *J* = 15.4 Hz, 1H), 4.68 – 4.61 (m, 1H), 3.07 (dd, *J* = 14.0, 5.2 Hz, 1H), 2.86 (dd, *J* = 14.0, 8.6 Hz, 1H), 2.21 – 2.11 (m, 5H), 1.44 (p, *J* = 7.1 Hz, 2H), 1.32 – 1.23 (m, 12H), 0.89 (t, *J* = 6.8 Hz, 3H) ppm.

¹³C{¹H}-NMR (126 MHz, CD_3OD) δ = 175.0, 168.5, 155.3, 146.4, 132.6, 128.8, 128.4, 125.4, 124.2, 115.5, 55.5, 37.7, 33.0, 30.8, 30.7, 30.5, 30.4, 30.3, 29.4, 23.7, 16.3, 14.5 ppm.

IR (ATR) $\tilde{\upsilon}$ = 3302 (m), 2923 (s), 2853 (s), 1720 (s), 1666 (s), 1610 (s), 1510 (s), 1461 (m), 1356 (m), 1264 (s), 1208 (s), 1120 (s), 977 (m), 820 (m), 579 (m) cm^{-1}.

HRMS (ESI) calcd for $C_{22}H_{34}NO_4$ $[M+H]^+$ 376.2482, found 376.2498.

(*S*,*E*)-2-(Hexadec-2-enamido)-3-(4-hydroxy-3-methylphenyl)propanoic acid (36)

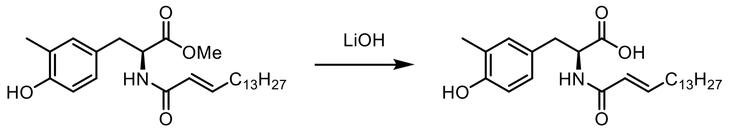

Following GP3: To a solution of methyl ester (43.1 mg, 0.097 mmol, 1.0 equiv.) in THF, MeOH and water (0.97 mL, 2:2:1) was added lithium hydroxide (57.9 mg, 2.42 mmol, 25 equiv.) to yield the amide (17.8 mg, 43%) as solid.

$[\alpha]_D^{20}$ = 22.7 (c = 0.23 MeOH)

^{1}H-NMR (500 MHz, CD_3OD) δ = 6.91 (s, 1H), 6.84 (dd, *J* = 8.2, 2.1 Hz, 1H), 6.74 (dt, *J* = 14.6, 7.0 Hz, 1H), 6.63 (d, *J* = 8.1 Hz, 1H), 5.95 (d, *J* = 15.3 Hz, 1H), 4.66 – 4.57 (m, 1H), 3.07 (dd, *J* = 14.0, 5.2 Hz, 1H), 2.86 (dd, *J* = 13.9, 8.5 Hz, 1H), 2.21 – 2.15 (m, 2H), 2.13 (s, 3H), 1.50 – 1.41 (m, 2H), 1.33 – 1.25 (m, 20H), 0.90 (t, *J* = 6.8 Hz, 3H) ppm.

^{13}C{^{1}H}-NMR (126 MHz, CD_3OD) δ = 175.1, 168.5, 155.4, 146.4, 132.6, 128.9, 128.4, 125.4, 124.3, 115.5, 55.6, 37.8, 33.1, 33.0, 30.8, 30.7, 30.6, 30.5, 30.3, 29.4, 23.8, 16.3, 14.5 ppm.

IR (ATR) $\tilde{\upsilon}$ = 3330 (m), 2916 (s), 2848 (s), 1724 (s), 1671 (m), 1604 (m), 1540 (m), 1462 (m), 1362 (m), 1267 (s), 1210 (s), 1117 (s), 974 (m), 810 (m), 575 (m) cm^{-1}.

HRMS (ESI) calcd for $C_{26}H_{42}NO_4$ $[M+H]^+$ 432.3108, found 432.3105.

Methyl dodecanoylglycinate (37)

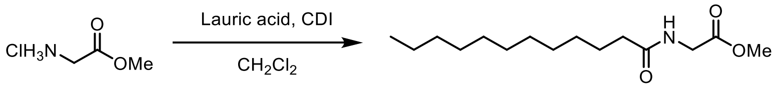

Following GP1: Lauric acid (1.00 g, 4.99 mmol, 1 equiv.), CDI (890 mg, 5.49 mmol, 1.1 equiv.) and methyl glycinate hydrochloride (689 mg, 5.49 mmol, 1.1 equiv.) in CH_2Cl_2 (25 mL) were used to yield the amide (1.13 g, 83%) as white solid.

^{1}H-NMR (500 MHz, $CDCl_3$) δ = 5.92 (s, br, 1H), 4.05 (d, J = 5.1 Hz, 2H), 3.76 (s, 3H), 2.24 (t, J = 7.6 Hz, 2H), 1.64 (p, J = 7.5 Hz, 2H), 1.25 (s, br, 16H), 0.88 (t, J = 6.9 Hz, 3H) ppm.

^{13}C{^{1}H}-NMR (126 MHz, $CDCl_3$) δ = 173.4, 170.8, 52.5, 41.3, 36.6, 32.1, 29.7, 29.6, 29.5, 29.4, 25.7, 22.8, 14.2 ppm.

HRMS (ESI) calcd for $C_{15}H_{30}NO_3$ $[M+H]^+$ 272.2220, found 272.2225.

The analytical data are in accordance with literature data.**[5]**

Dodecanoyl glycine (38)

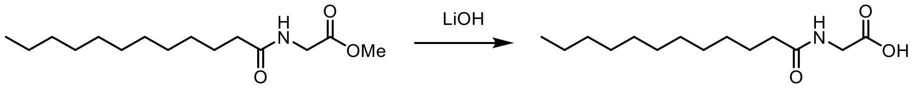

Following GP3: To a solution of methyl ester (2.00 g, 7.37 mmol, 1.0 equiv.) in THF, MeOH and water (90 mL, 2:2:1) was added lithium hydroxide (4.41 g, 184 mmol, 25 equiv.) to yield the amide (1.80 g, 95%) as white solid.

^{1}H-NMR (500 MHz, CD_3OD) δ = 3.89 (s, 2H), 2.25 (t, J = 7.5 Hz, 2H), 1.62 (p, J = 7.4 Hz, 2H), 1.36 – 1.27 (m, 16H), 0.90 (t, J = 6.8 Hz, 3H) ppm.

^{13}C{^{1}H}-NMR (126 MHz, CD_3OD) δ = 176.7, 173.1, 41.7, 36.8, 33.1, 30.8, 30.6, 30.5, 30.3, 26.9, 23.8, 14.5 ppm.

HRMS (ESI) calcd for $C_{14}H_{28}NO_3$ $[M+H]^+$ 258.2064, found 258.2074.

Supplementary Table 2. Comparison of chemical shifts of isolated and synthetic stieleriacine C (solvent DMSO-d6)

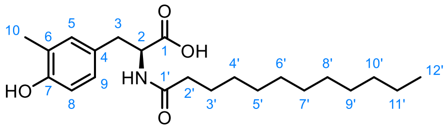

N°	isolated [700 MHz, 176 MHz]		synthesized (34) 864 [500 MHz, 126 MHz]			
	δ H [ppm]	δ C [ppm]	δ H [ppm]	δ C [ppm]	$|\Delta\delta_H|$ *[ppm]*	$|\Delta\delta_C|$ *[ppm]*
**1**		173.3		173.5		0.2
**2**	4.26 (m)	53.9	4.25 (m)	54.1	0.01	0.2
**3**	2.87 (dd, 13.8, 5.0)<br>2.68 (dd, 13.8, 9.1)	36.1	2.87 (dd, 13.8, 4.8)<br>2.67 (dd, 13.8, 9.1)	36.2	0.00<br>0.01	0.1
**4**		127.8		128.0		0.2
**5**	6.86 (s)	131.3	6.86 (s)	131.4	0.00	0.1
**6**		123.1		123.2		0.1
**7**		153.8		153.9		0.1
**8**	6.62 (d, 8.0)	114.2	6.62 (d, 8.1)	114.3	0.00	0.1
**9**	6.79 (dd, 8.2, 1.8)	127.1	6.79 (dd, 8.2, 2.2)	127.3	0.00	0.2
**10**	2.05 (s)	16.0	2.04 (s)	16.1	0.01	0.1
**1'**		171.9		172.1		0.2
**2'**	2.02 (td, 7.3, 2.5)	35.2	2.01 (m)	35.3	0.01	0.1
**3'**	1.39 (m)	25.2	1.38 (m)	25.4	0.01	0.2
**4'**	1.14 (m)	28.5	1.13 (m)	28.6	0.01	0.1
**5'-11'**	1.22 (m)	29.0-28.7	1.21 (m)	29.2-28.8	0.01	0.2-0.1
**10'**		31.3		31.4		0.1
**11'**		22.1		22.2		0.1
**12'**	0.85 (t, 7.0)	13.9	0.85 (t, 6.7)	14.1	0.00	0.2
**NH**	7.86 (s, br)		7.88 (d, br, 7.7)		0.02	
**-OH**	9.02 (s, br)		9.08 (s, br)		0.06	

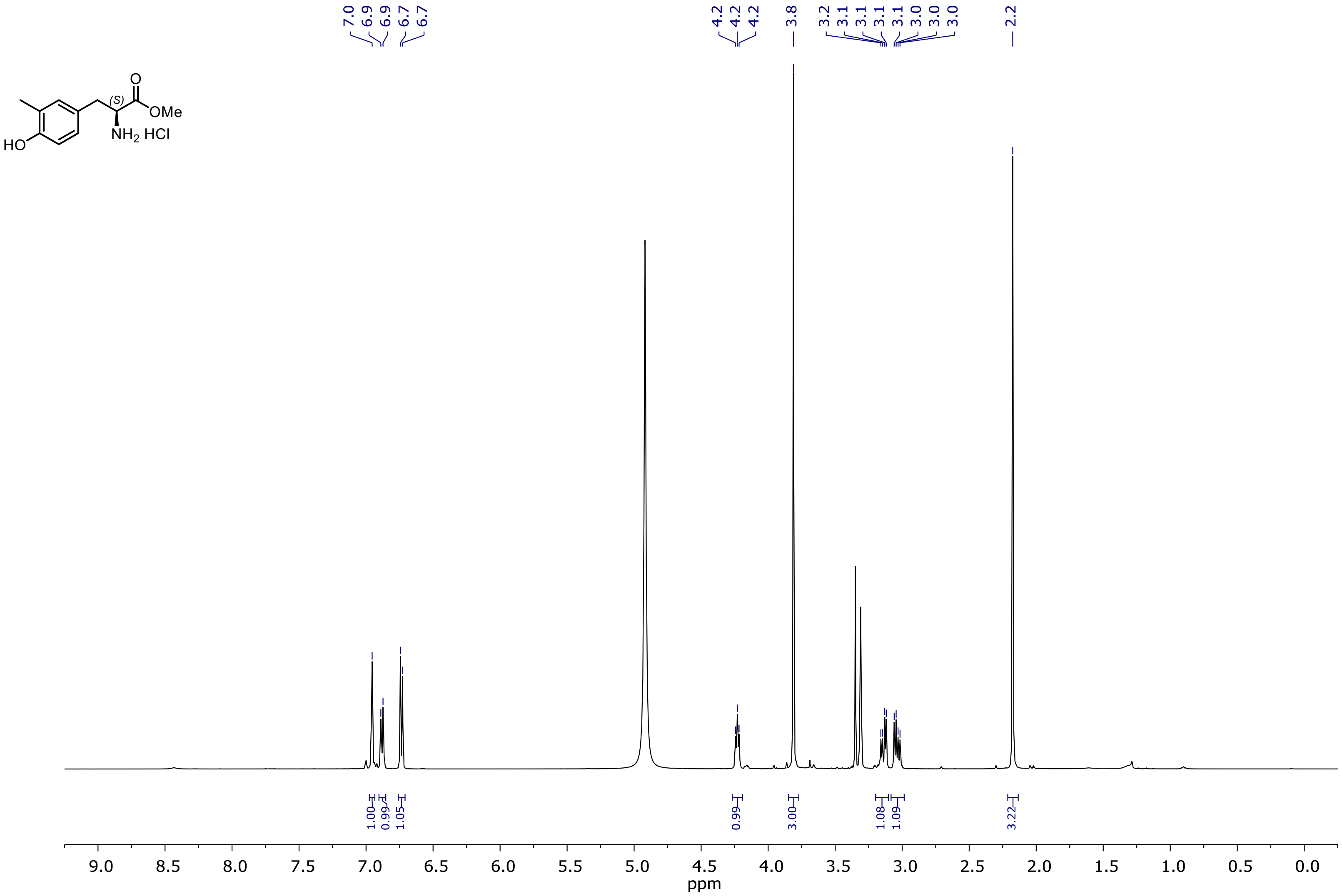

Supplementary Figure 10. ^{1}H-NMR (500 MHz, CD_3OD) of methyl (*S*)-2-amino-3-(4-hydroxy-3-methylphenyl)propanoate hydrochloride.

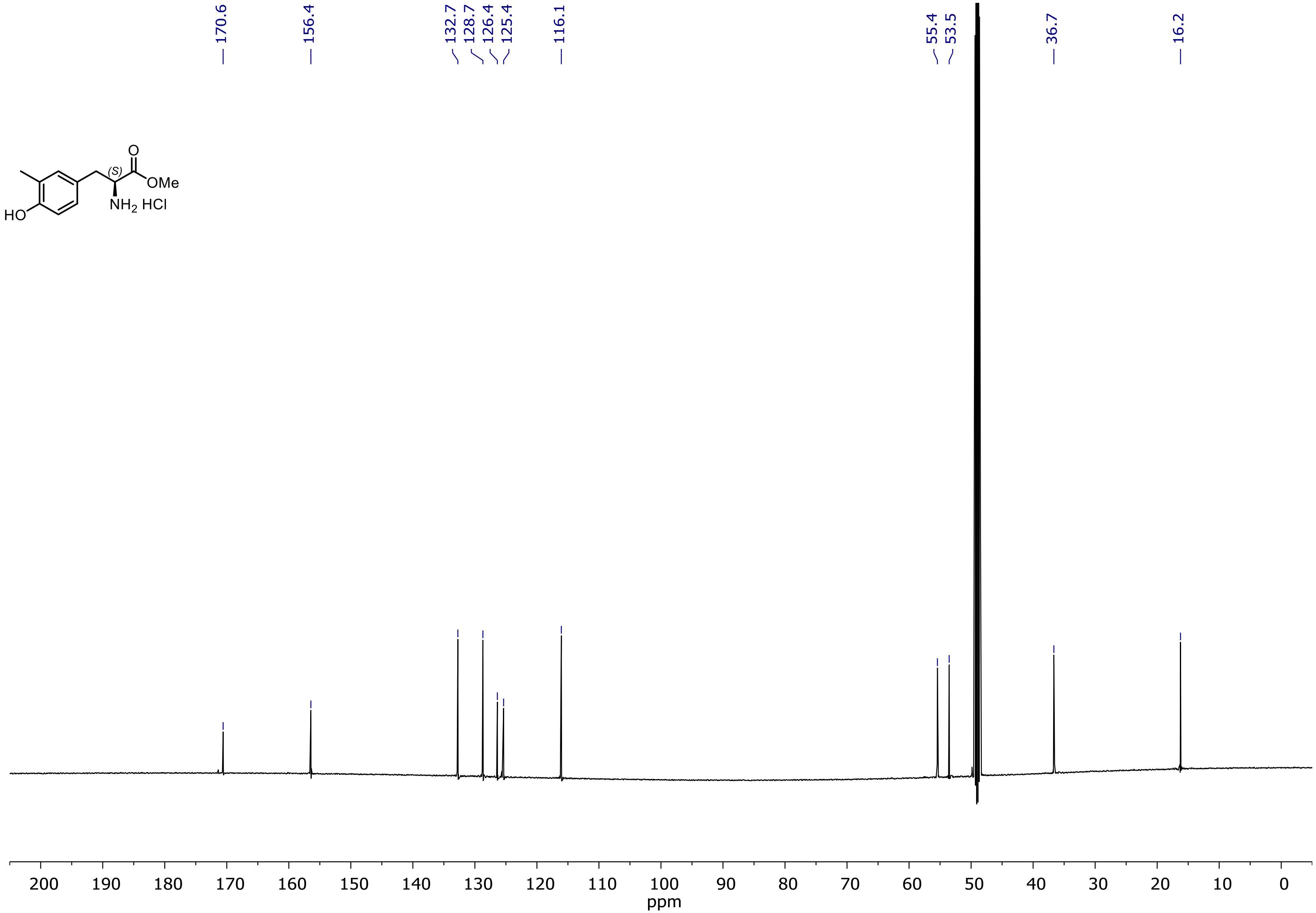

Supplementary Figure 11. $^{13}C\{^{1}H\}$-NMR (126 MHz, CD_3OD) of methyl (S)-2-amino-3-(4-hydroxy-3-methylphenyl)propanoate hydrochloride.

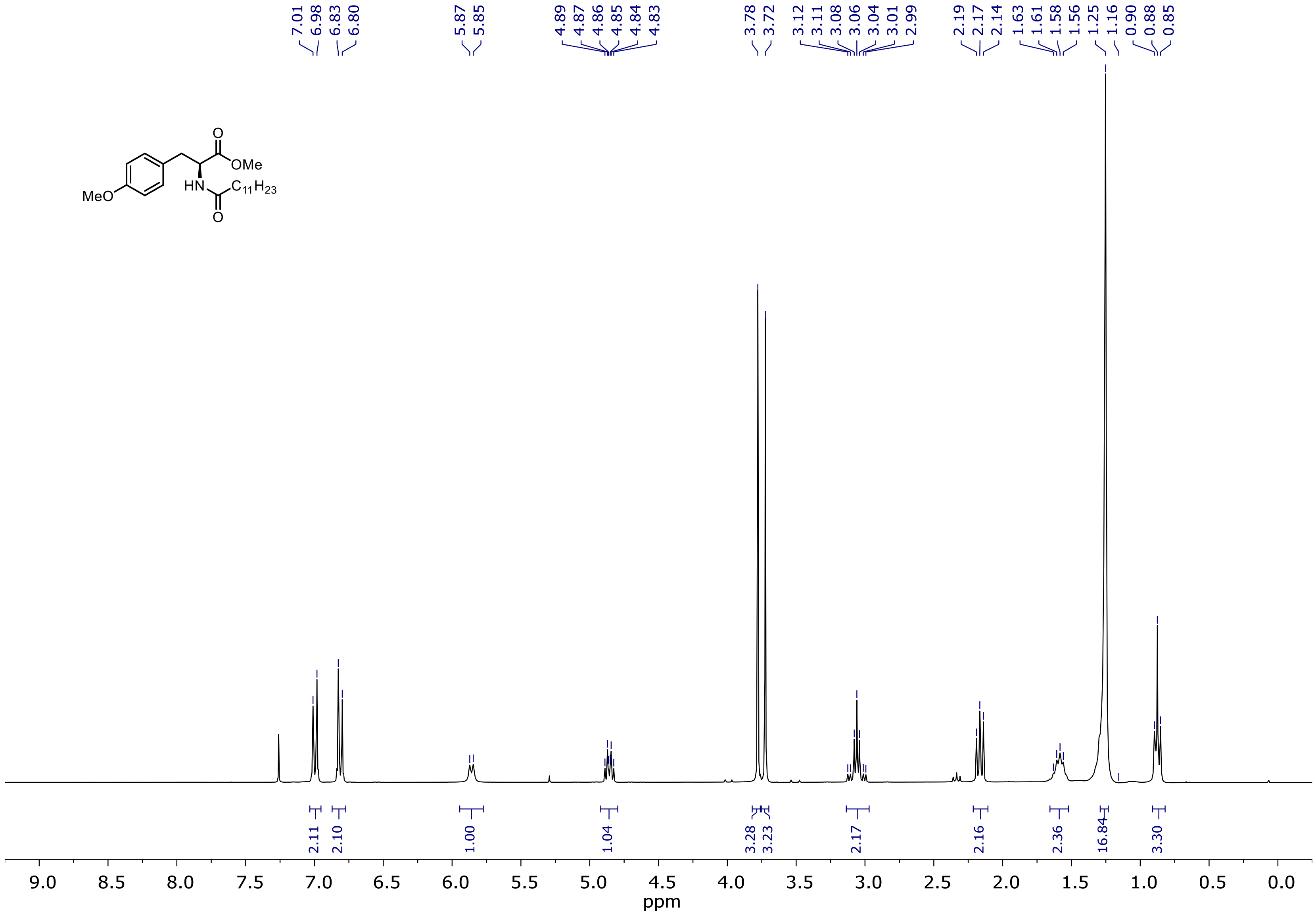

Supplementary Figure 12. ^{1}H-NMR (300 MHz, $CDCl_3$) of methyl (*S*)-2-dodecanamido-3-(4-methoxyphenyl)propanoate**.**

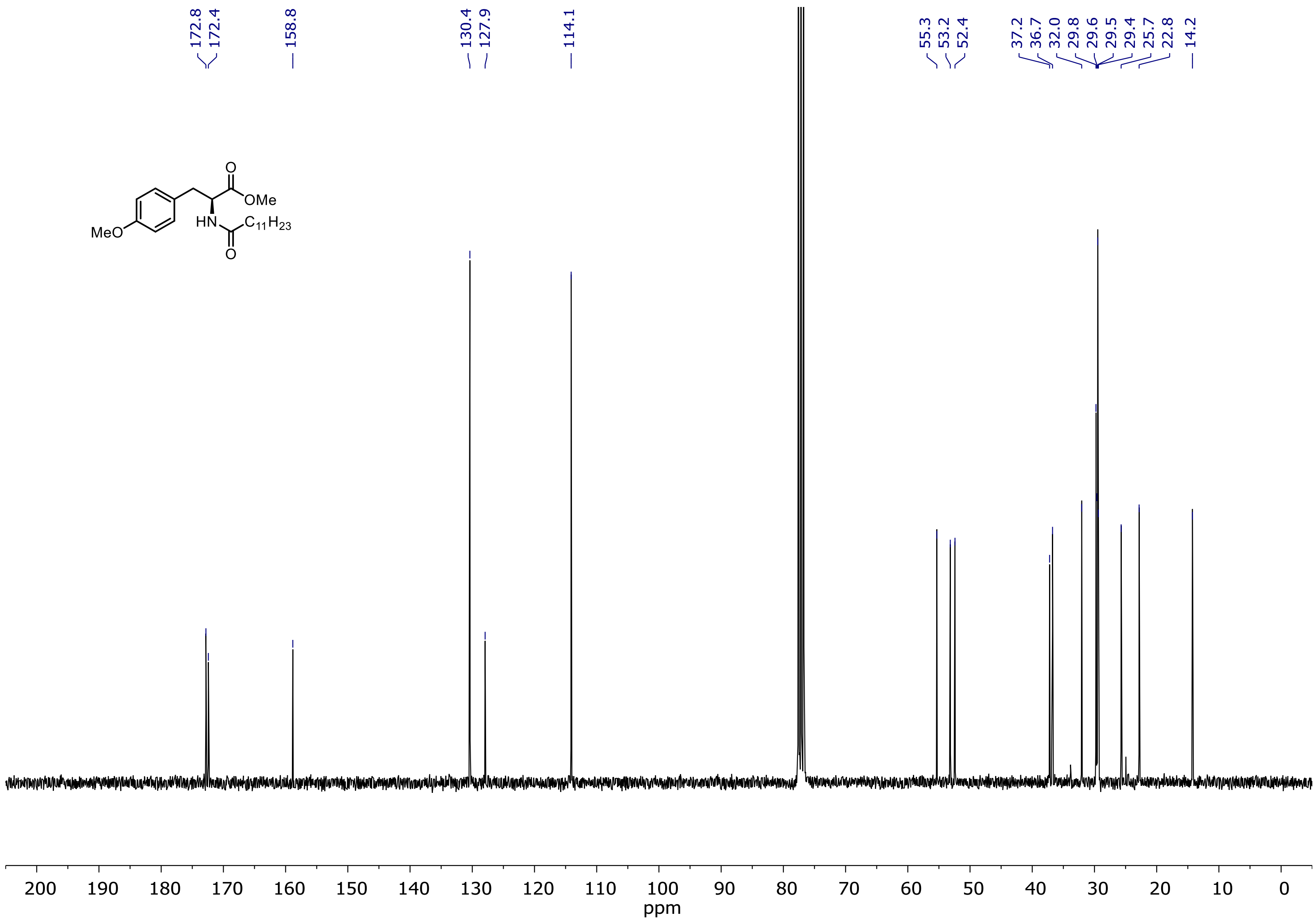

Supplementary Figure 13. $^{13}C\{^{1}H\}$-NMR (75 MHz, $CDCl_3$) of methyl (*S*)-2-dodecanamido-3-(4-methoxyphenyl)propanoate**.**

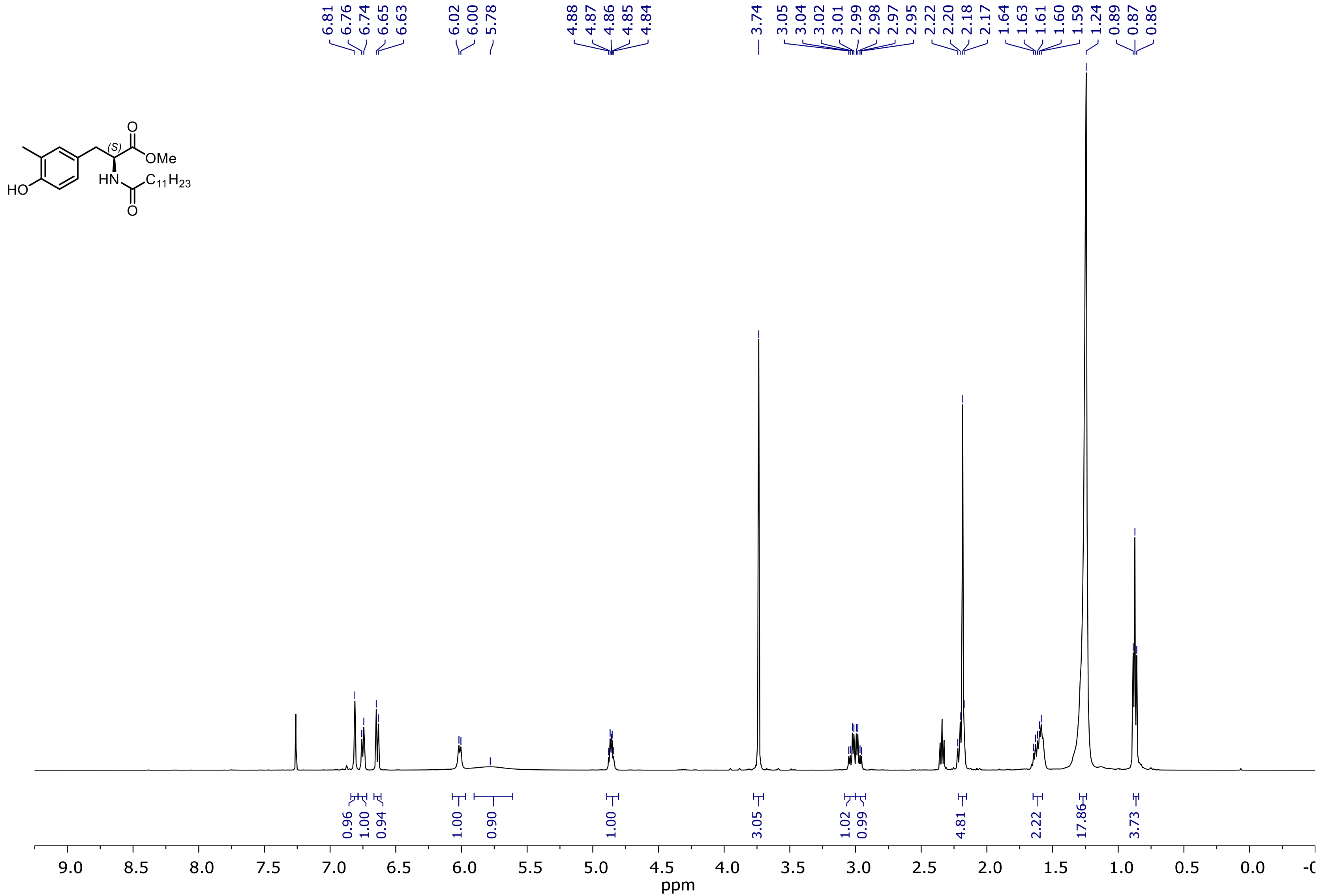

Supplementary Figure 14. ^{1}H-NMR (500 MHz, $CDCl_3$) of methyl (S)-2-dodecanamido-3-(4-hydroxy-3-methylphenyl)propanoate**.**

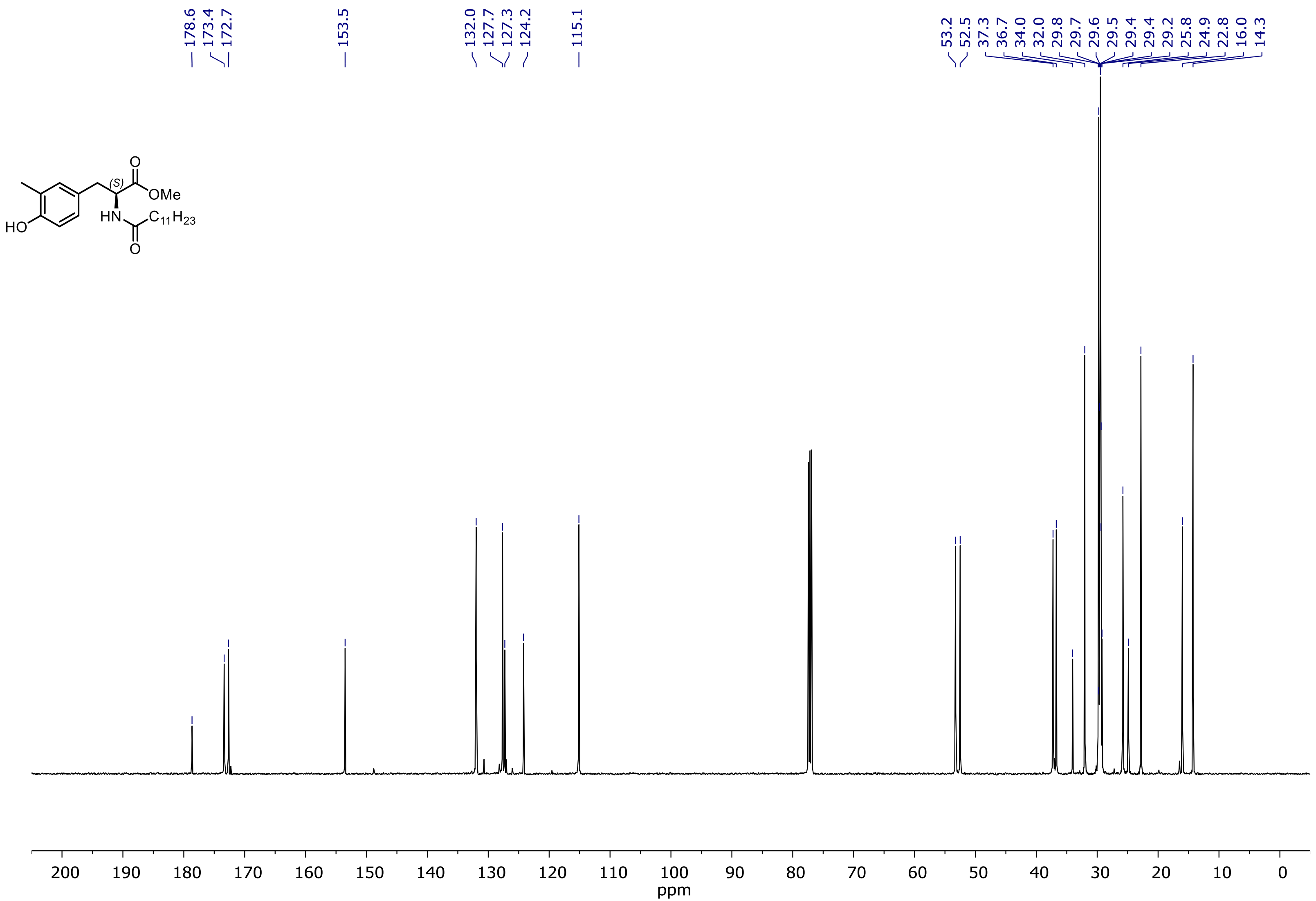

Supplementary Figure 15. $^{13}C\{^{1}H\}$-NMR (126 MHz, $CDCl_3$) of methyl (S)-2-dodecanamido-3-(4-hydroxy-3-methylphenyl)propanoate**.**

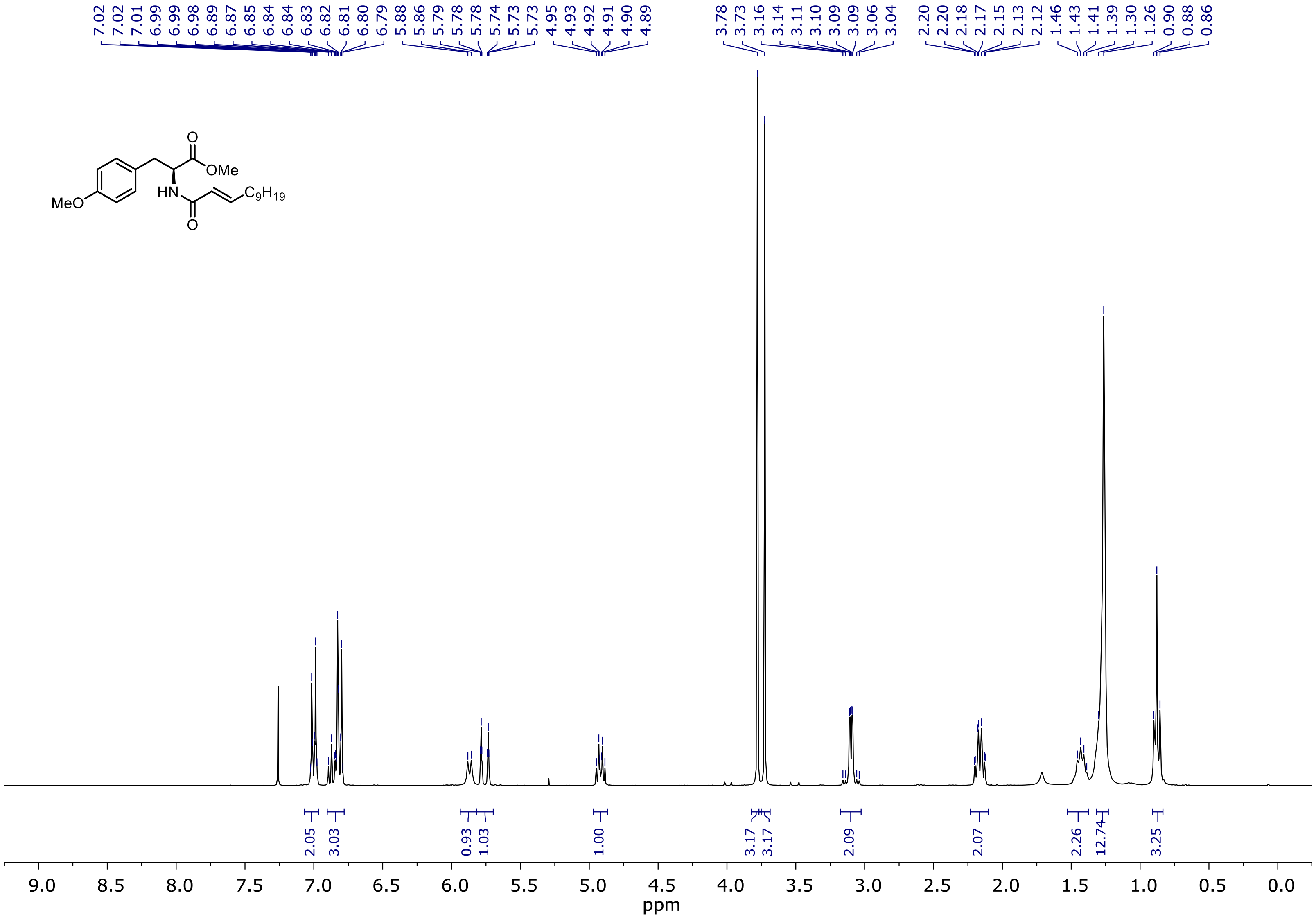

Supplementary Figure 16. ^{1}H-NMR (300 MHz, $CDCl_3$) of methyl (*S*,*E*)-2-(dodec-2-enamido)-3-(4-methoxyphenyl)propanoate.

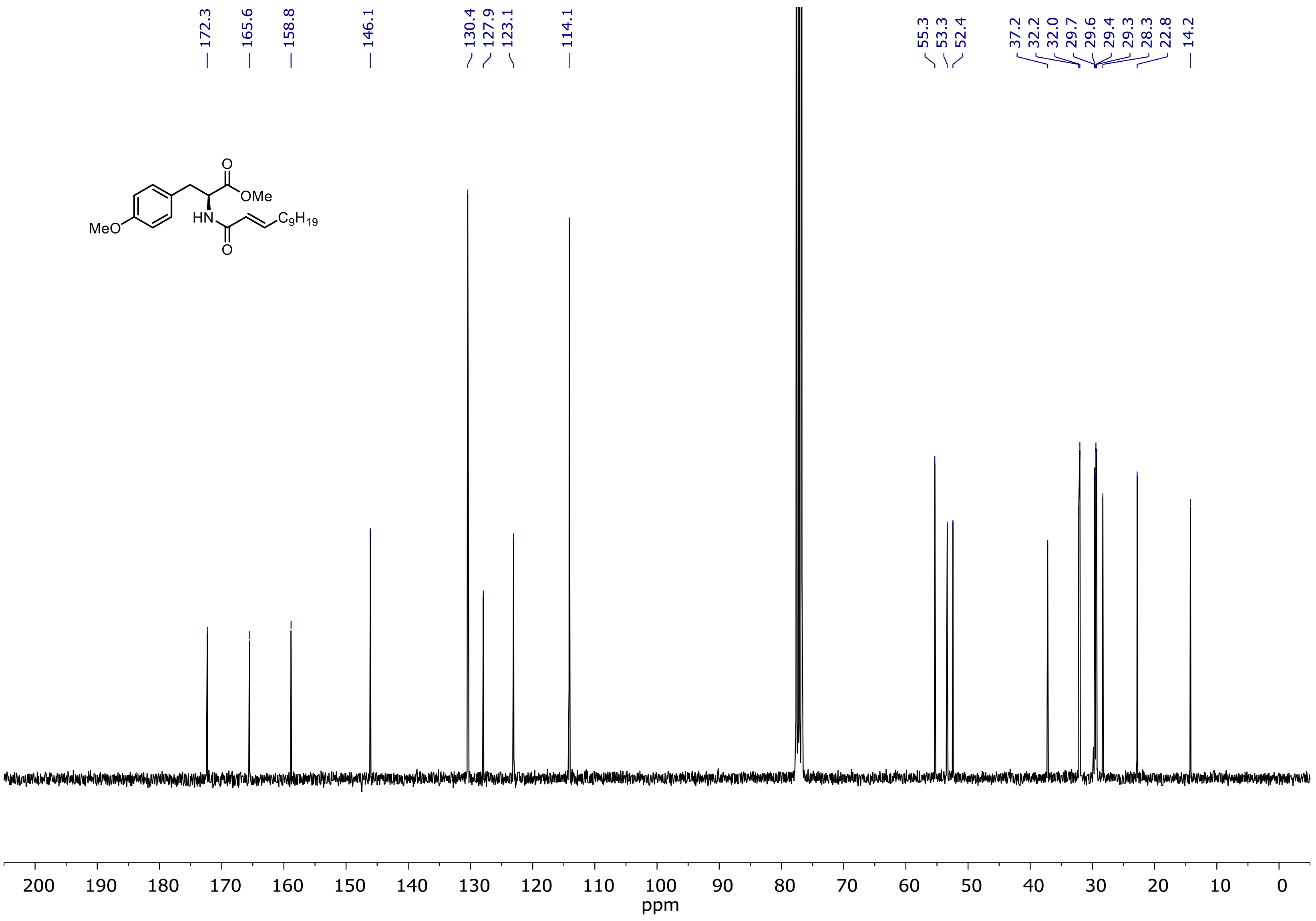

Supplementary Figure 17. $^{13}C\{^1H\}$-NMR (75 MHz, $CDCl_3$) of methyl (*S*,*E*)-2-(dodec-2-enamido)-3-(4-methoxyphenyl)propanoate.

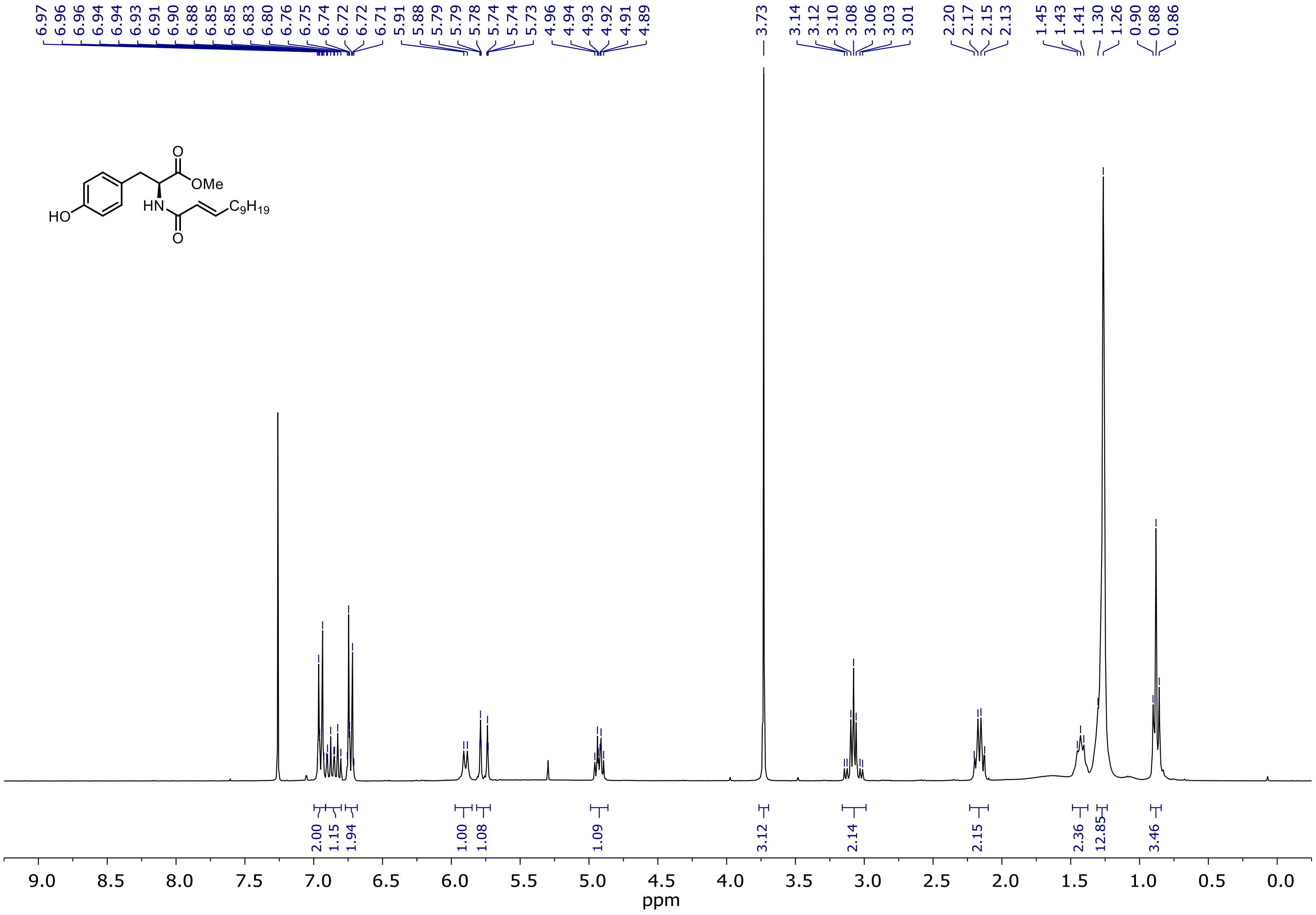

Supplementary Figure 18. ^{1}H-NMR (300 MHz, $CDCl_3$) of methyl *(E)*-dodec-2-enoyl-*L*-tyrosinate**.**

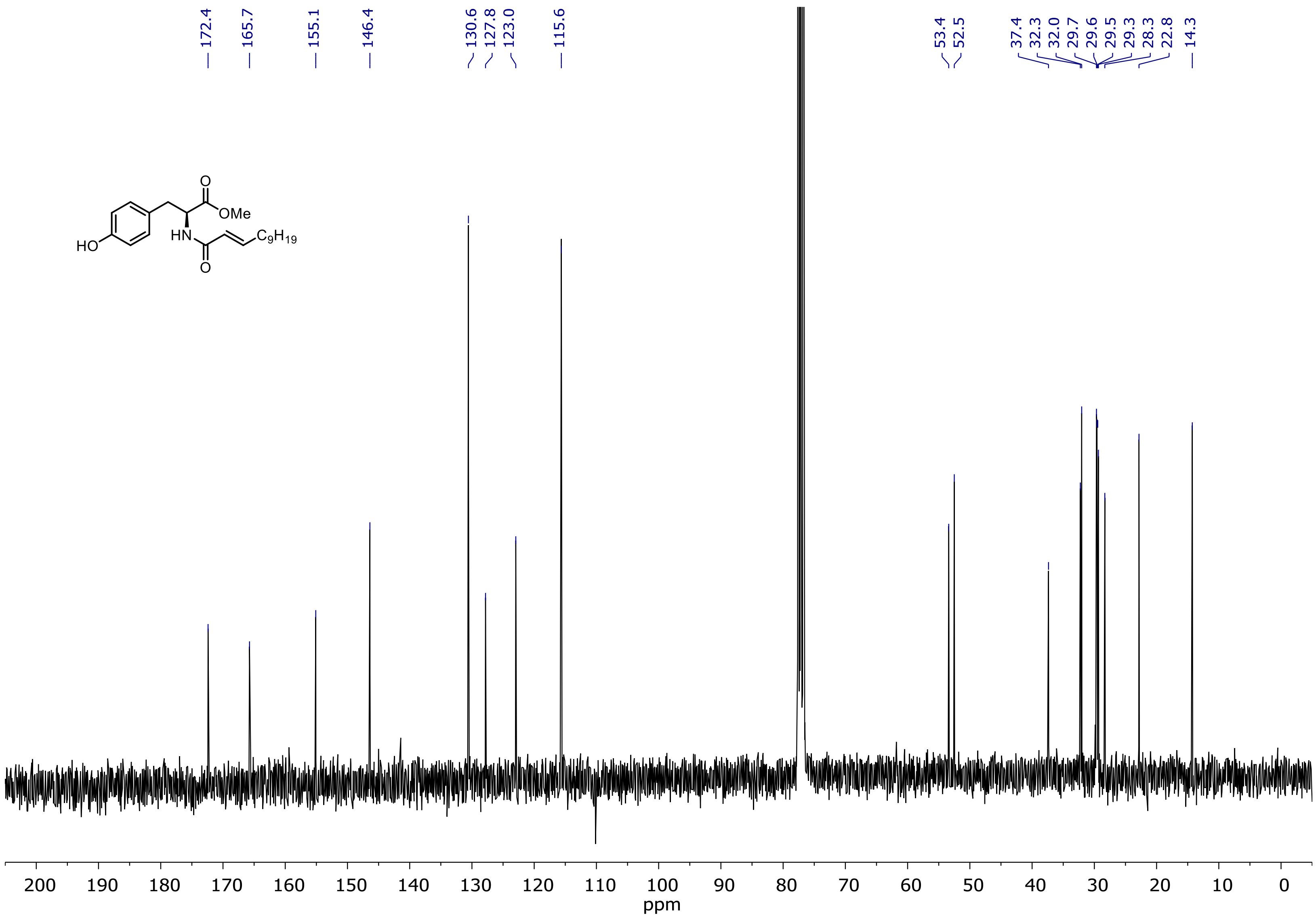

Supplementary Figure 19. $^{13}C\{^{1}H\}$-NMR (75 MHz, $CDCl_3$) of methyl (*E*)-dodec-2-enoyl-*L*-tyrosinate.

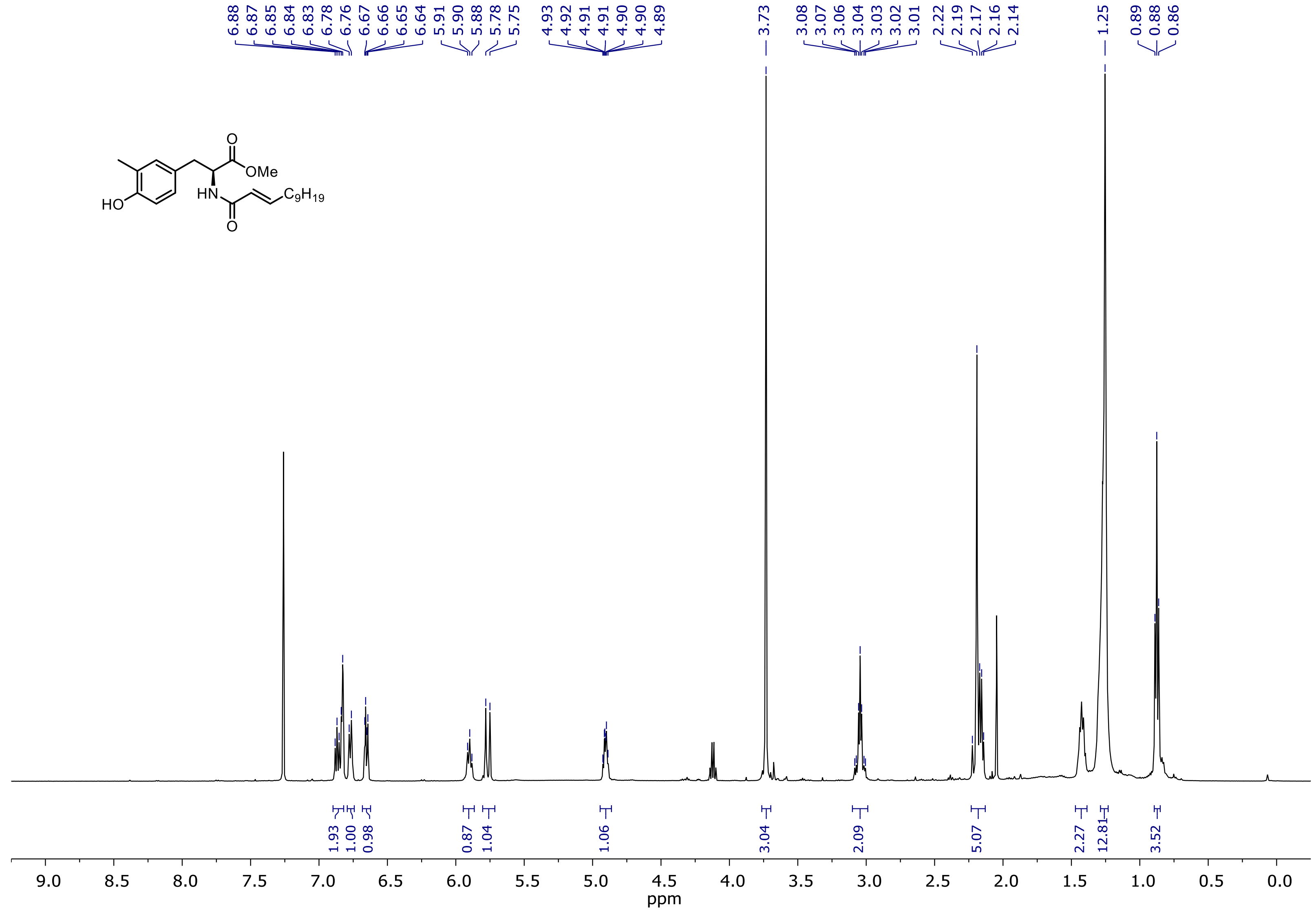

Supplementary Figure 20. ^{1}H-NMR (500 MHz, $CDCl_3$) of methyl (*S*,*E*)-2-(dodec-2-enamido)-3-(4-hydroxy-3-methylphenyl)propanoate.

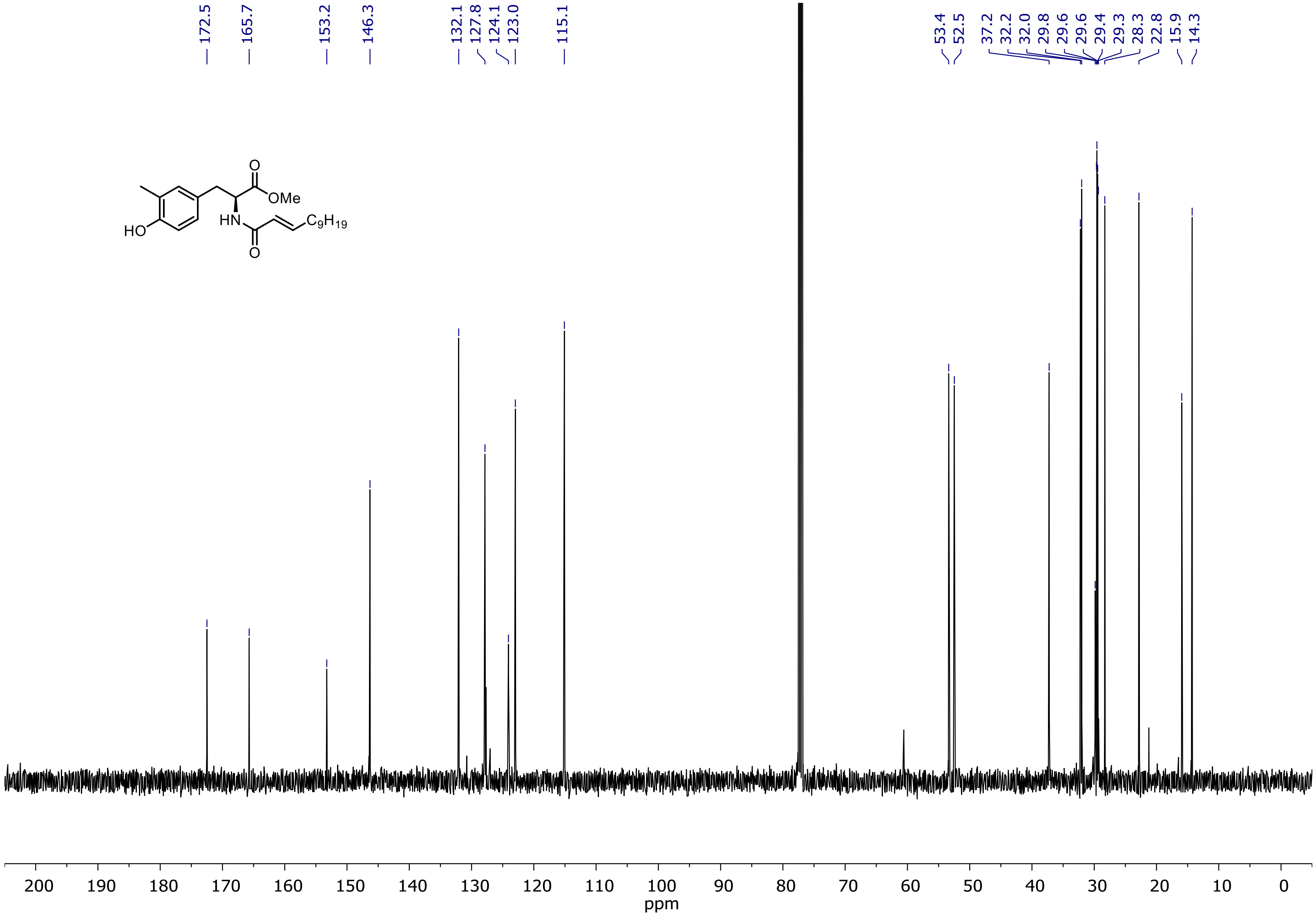

Supplementary Figure 21. $^{13}C\{^{1}H\}$-NMR (126 MHz, $CDCl_3$) of methyl (*S*,*E*)-2-(dodec-2-enamido)-3-(4-hydroxy-3-methylphenyl)propanoate.

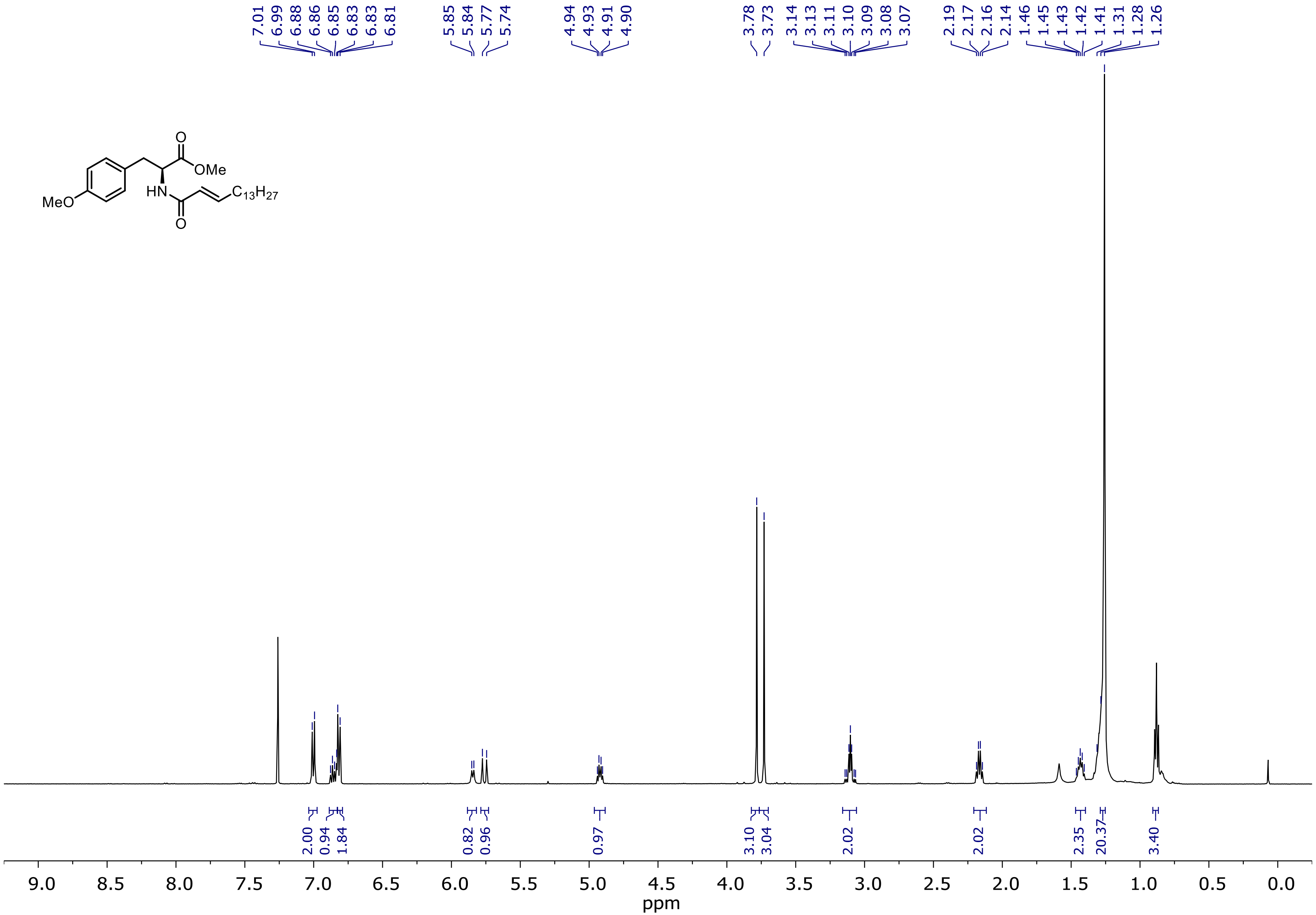

Supplementary Figure 22. ^{1}H-NMR (500 MHz, $CDCl_3$) of methyl (*S*,*E*)-2-(hexadec-2-enamido)-3-(4-methoxyphenyl)propanoate**.**

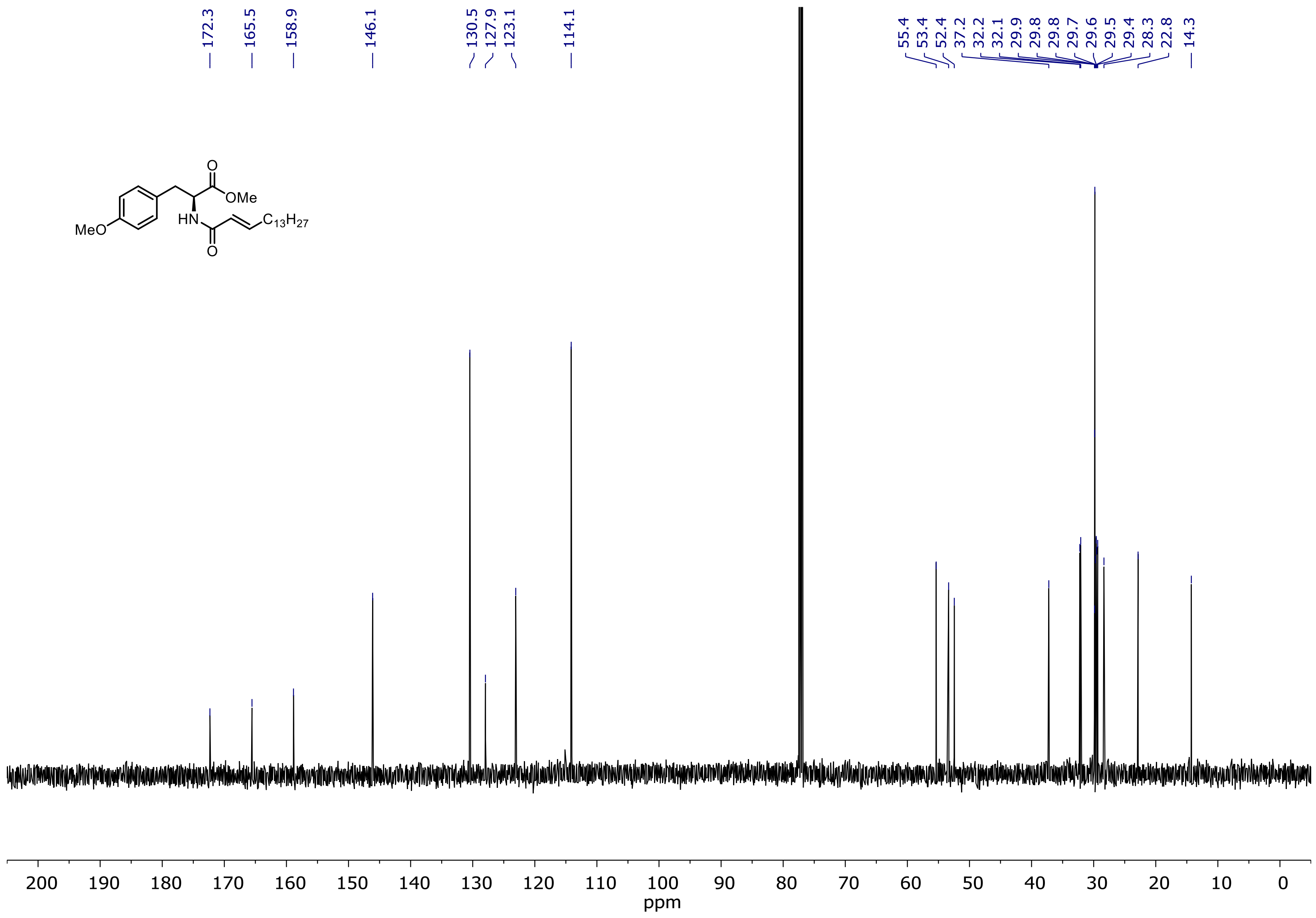

Supplementary Figure 23. $^{13}C\{^{1}H\}$-NMR (126 MHz, $CDCl_3$) of methyl (*S*,*E*)-2-(hexadec-2-enamido)-3-(4-methoxyphenyl)propanoate**.**

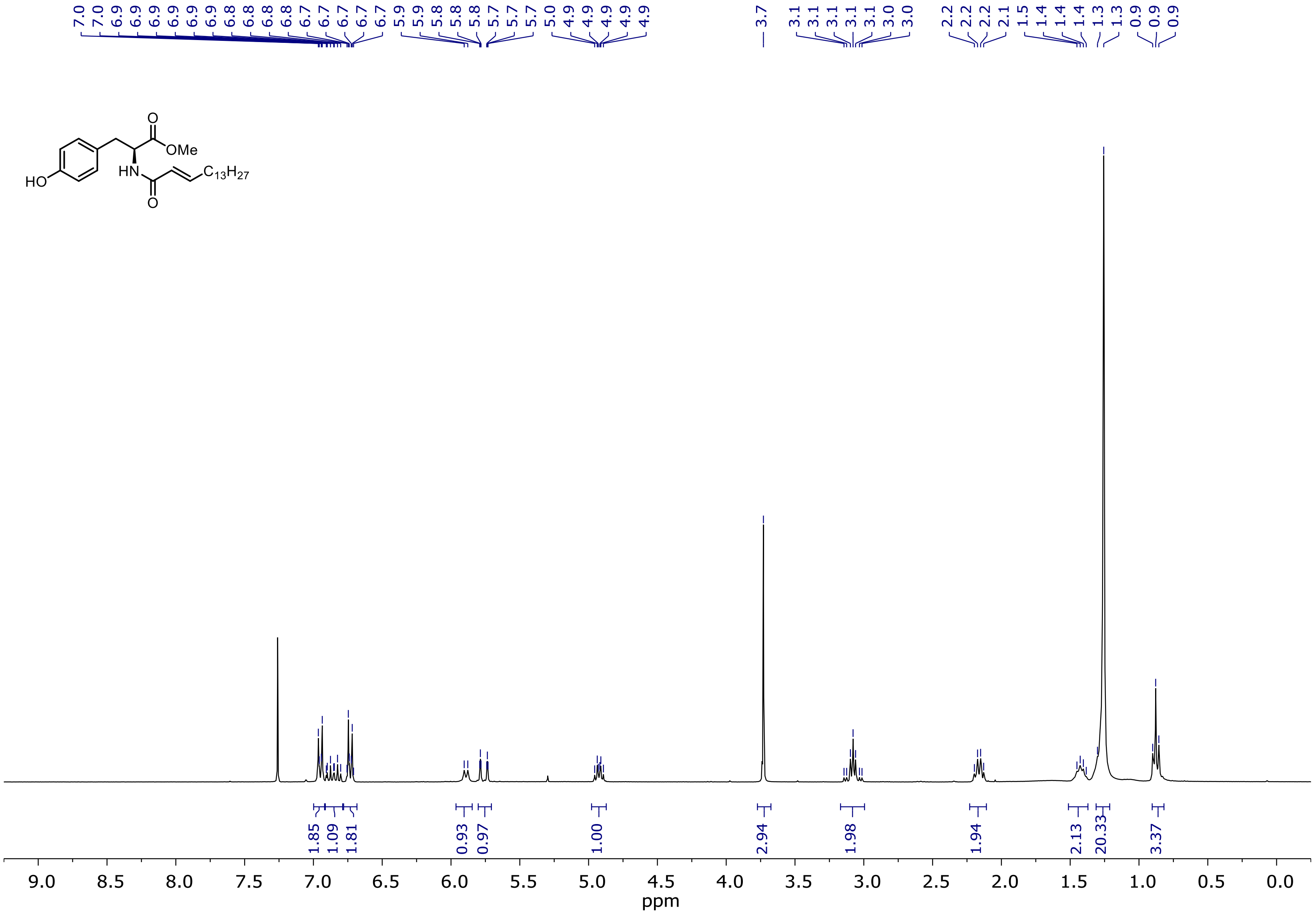

Supplementary Figure 24. ^{1}H-NMR (300 MHz, $CDCl_3$) of methyl (*E*)-hexadec-2-enoyl-*L*-tyrosinate.

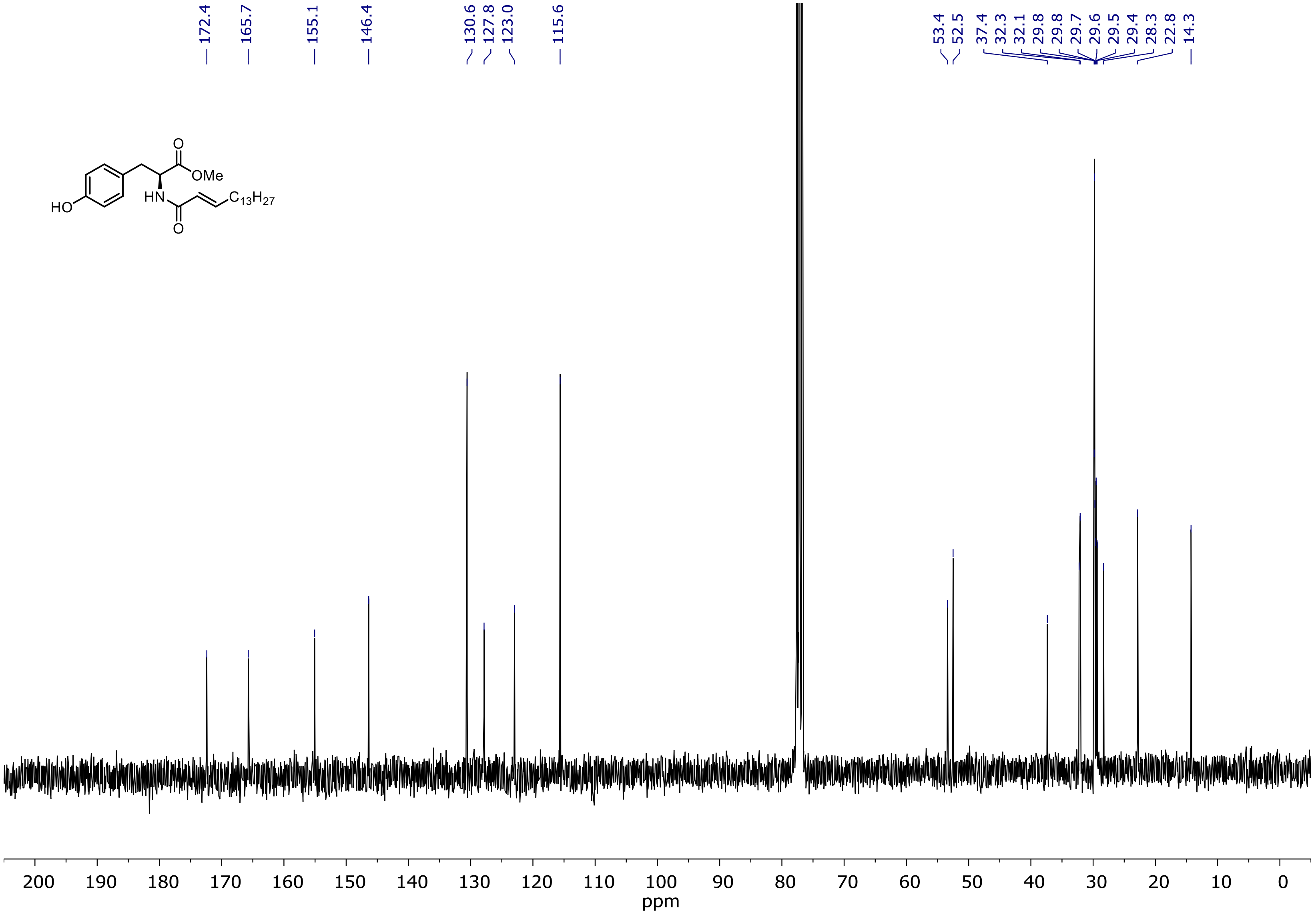

Supplementary Figure 25. $^{13}C\{^1H\}$-NMR (75 MHz, $CDCl_3$) of methyl (*E*)-hexadec-2-enoyl-*L*-tyrosinate.

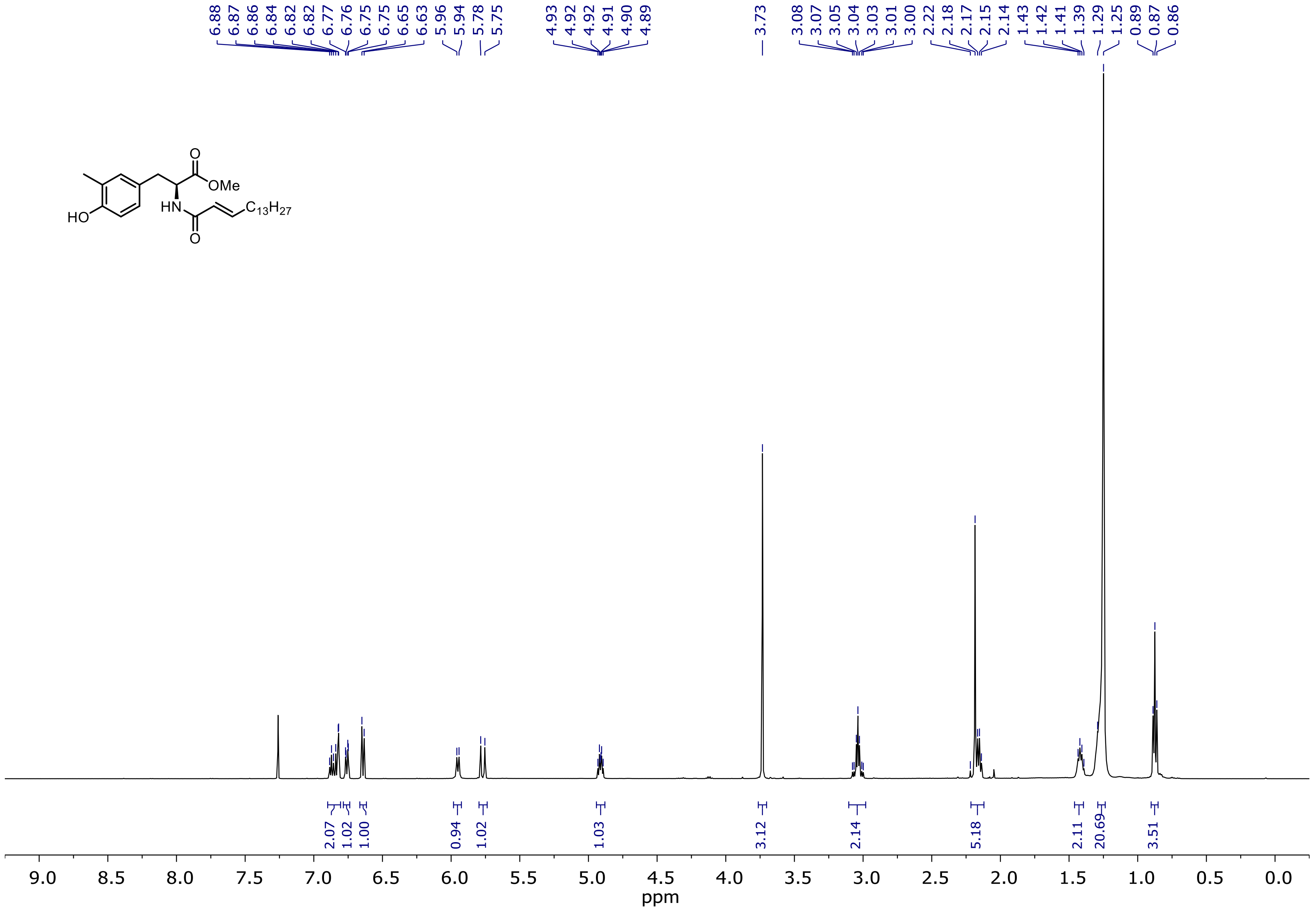

Supplementary Figure 26. ^{1}H-NMR (500 MHz, $CDCl_3$) of methyl (*S*,*E*)-2-(hexadec-2-enamido)-3-(4-hydroxy-3-methylphenyl)propanoate.

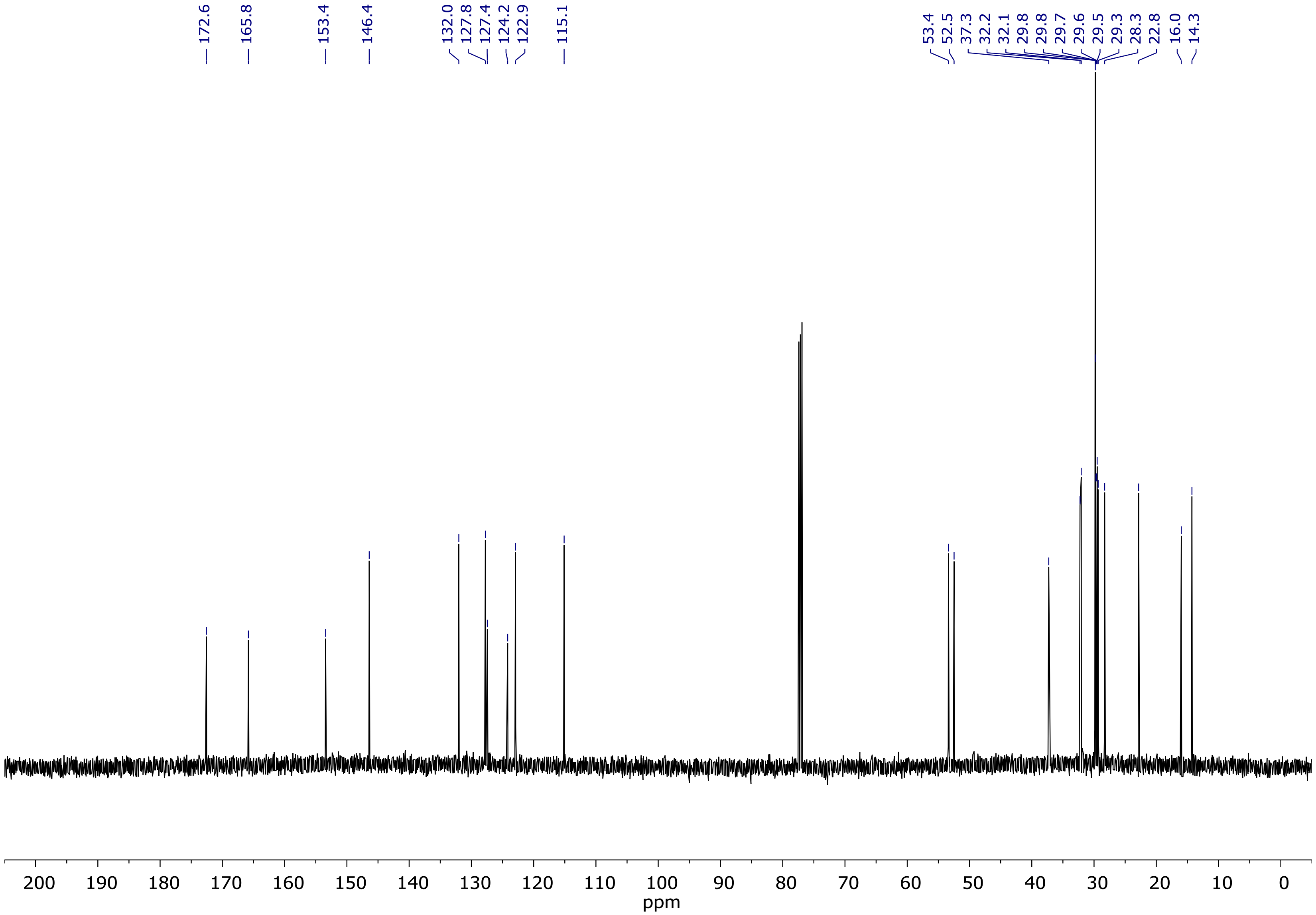

Supplementary Figure 27. $^{13}C\{^{1}H\}$-NMR (126 MHz, $CDCl_3$) of methyl (*S*,*E*)-2-(hexadec-2-enamido)-3-(4-hydroxy-3-methylphenyl)propanoate**.**

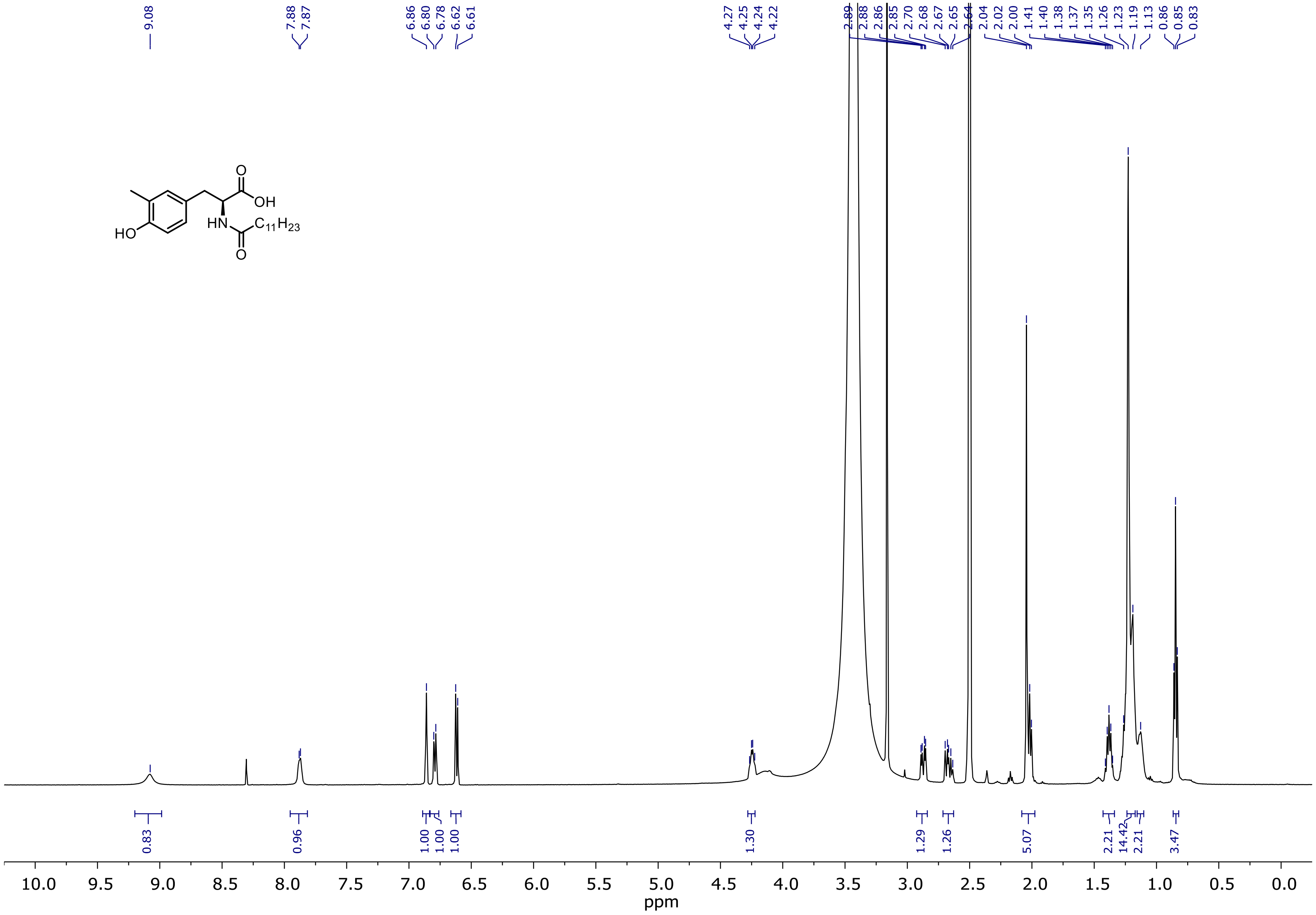

Supplementary Figure 28. ^{1}H-NMR (500 MHz, DMSO-d6) of (*S*)-2-dodecanamido-3-(4-hydroxy-3-methylphenyl)propanoic acid**.**

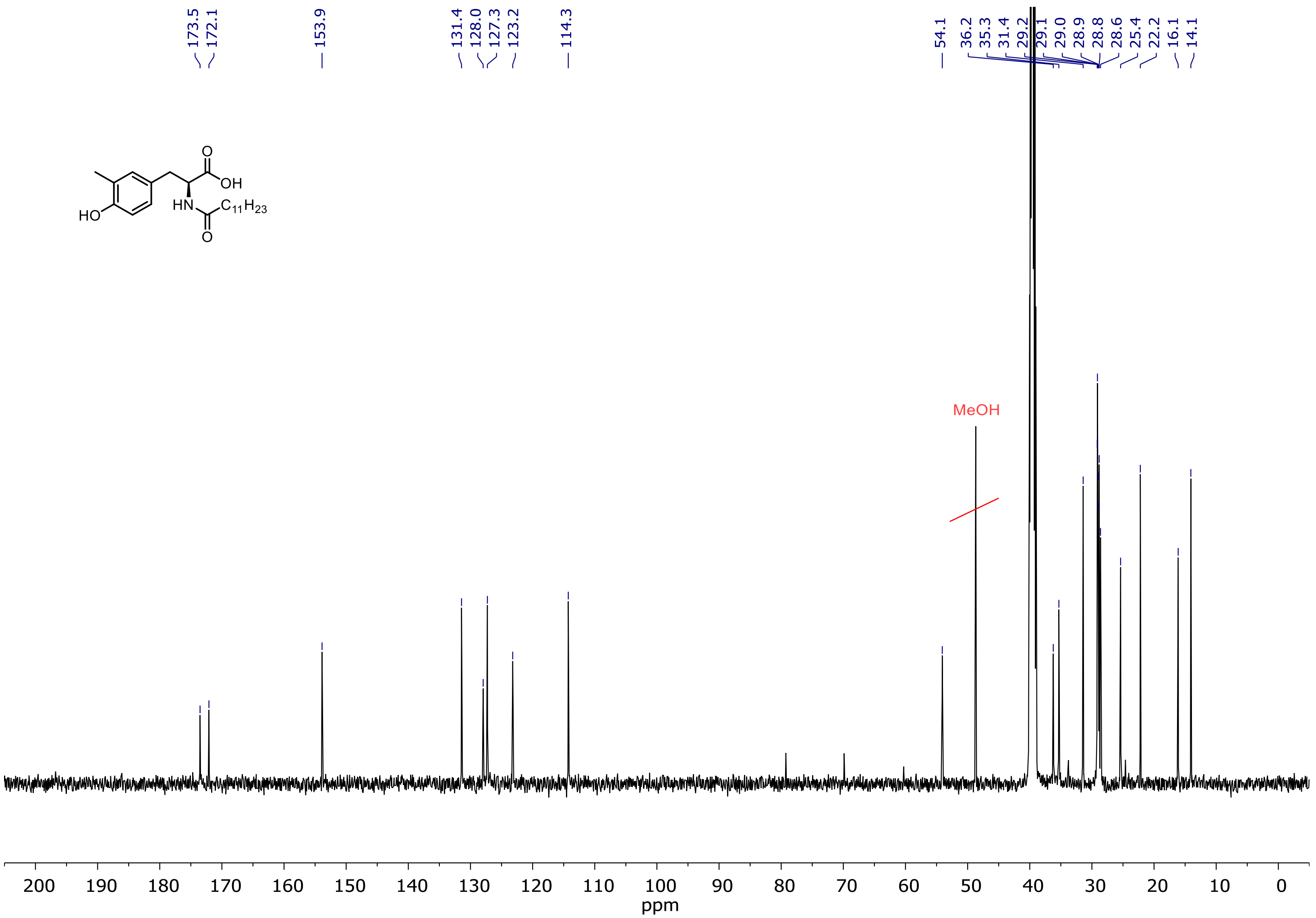

Supplementary Figure 29. $^{13}C\{^{1}H\}$-NMR (126 MHz, DMSO-d6) of (*S*)-2-dodecanamido-3-(4-hydroxy-3-methylphenyl)propanoic acid**.**

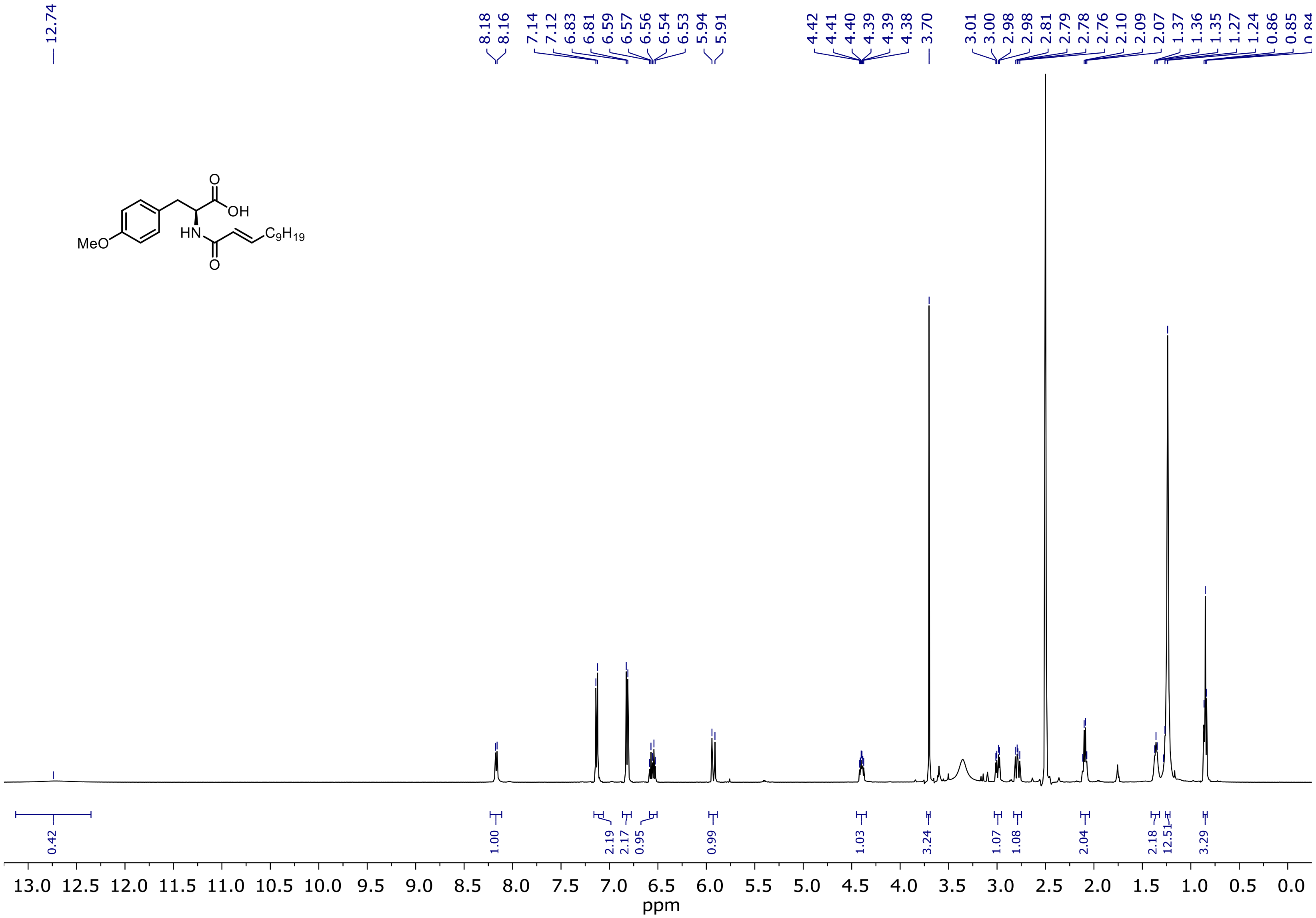

Supplementary Figure 30. ^{1}H-NMR (500 MHz, DMSO-d6) of (*S*,*E*)-2-(dodec-2-enamido)-3-(4-methoxyphenyl)propanoic acid.

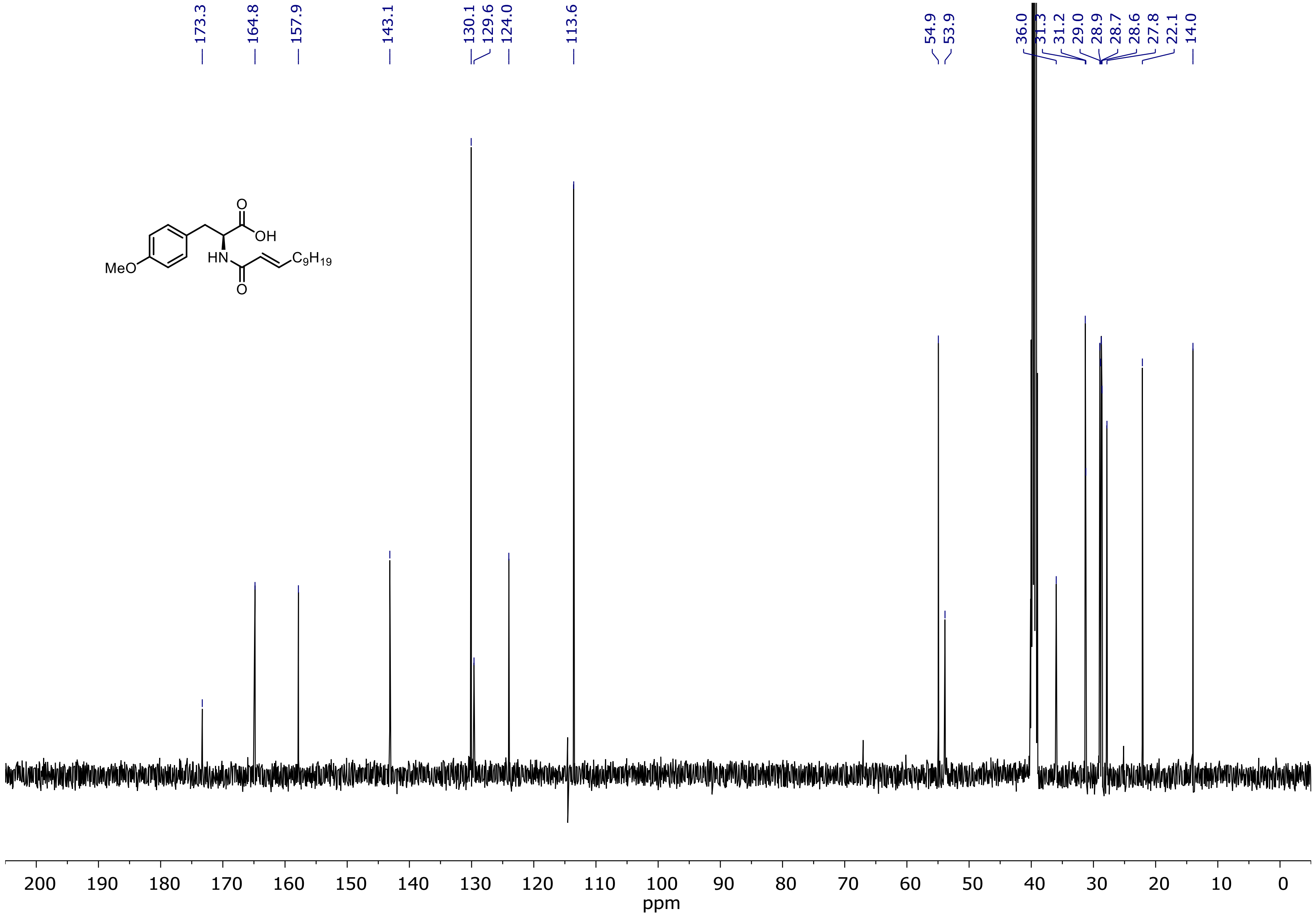

Supplementary Figure 31. $^{13}C\{^{1}H\}$-NMR (126 MHz, DMSO-d6) of (*S*,*E*)-2-(dodec-2-enamido)-3-(4-methoxyphenyl)propanoic acid.

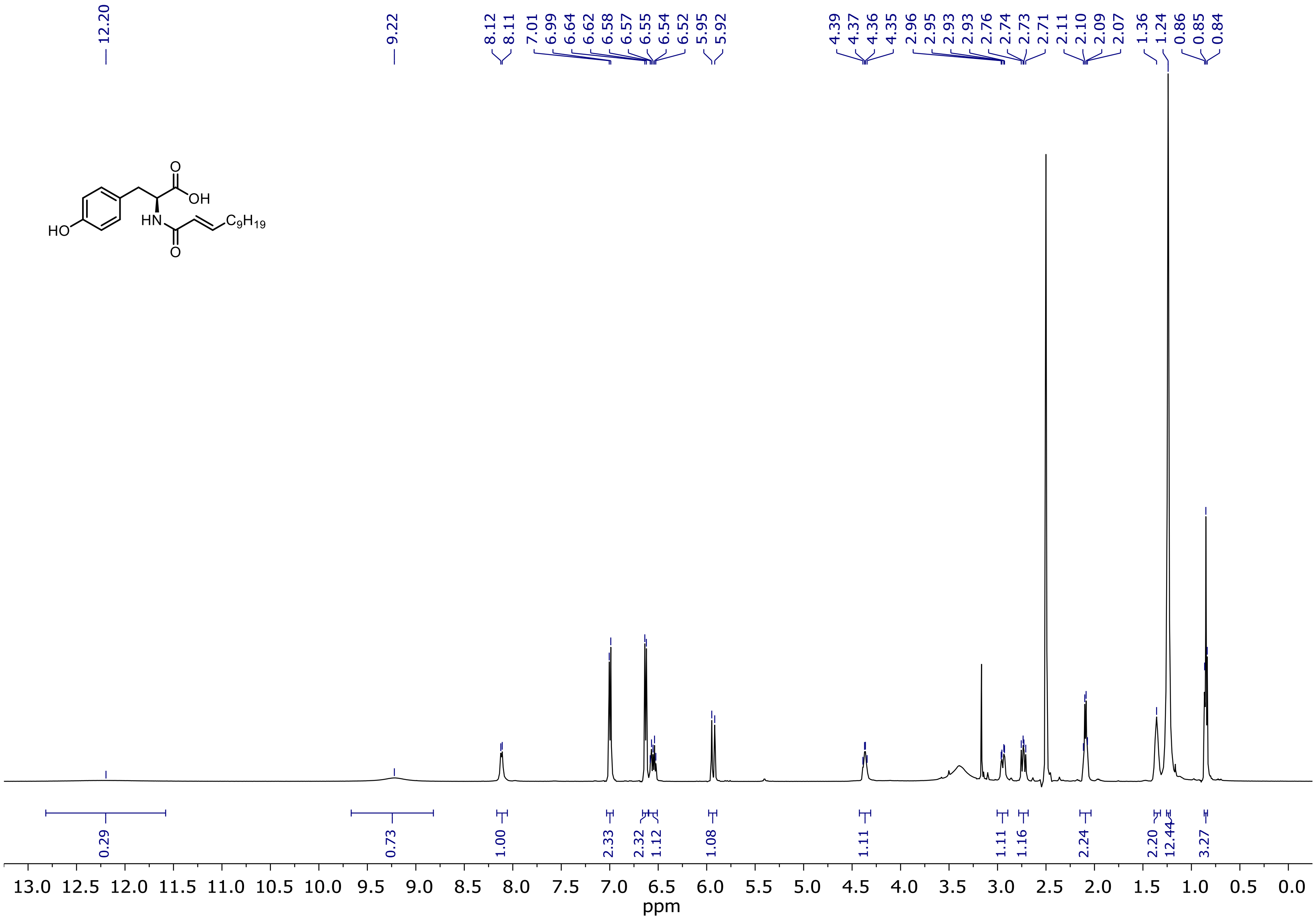

Supplementary Figure 32. ^{1}H-NMR (500 MHz, DMSO-d6) of (*E*)-dodec-2-enoyl-*L*-tyrosine.

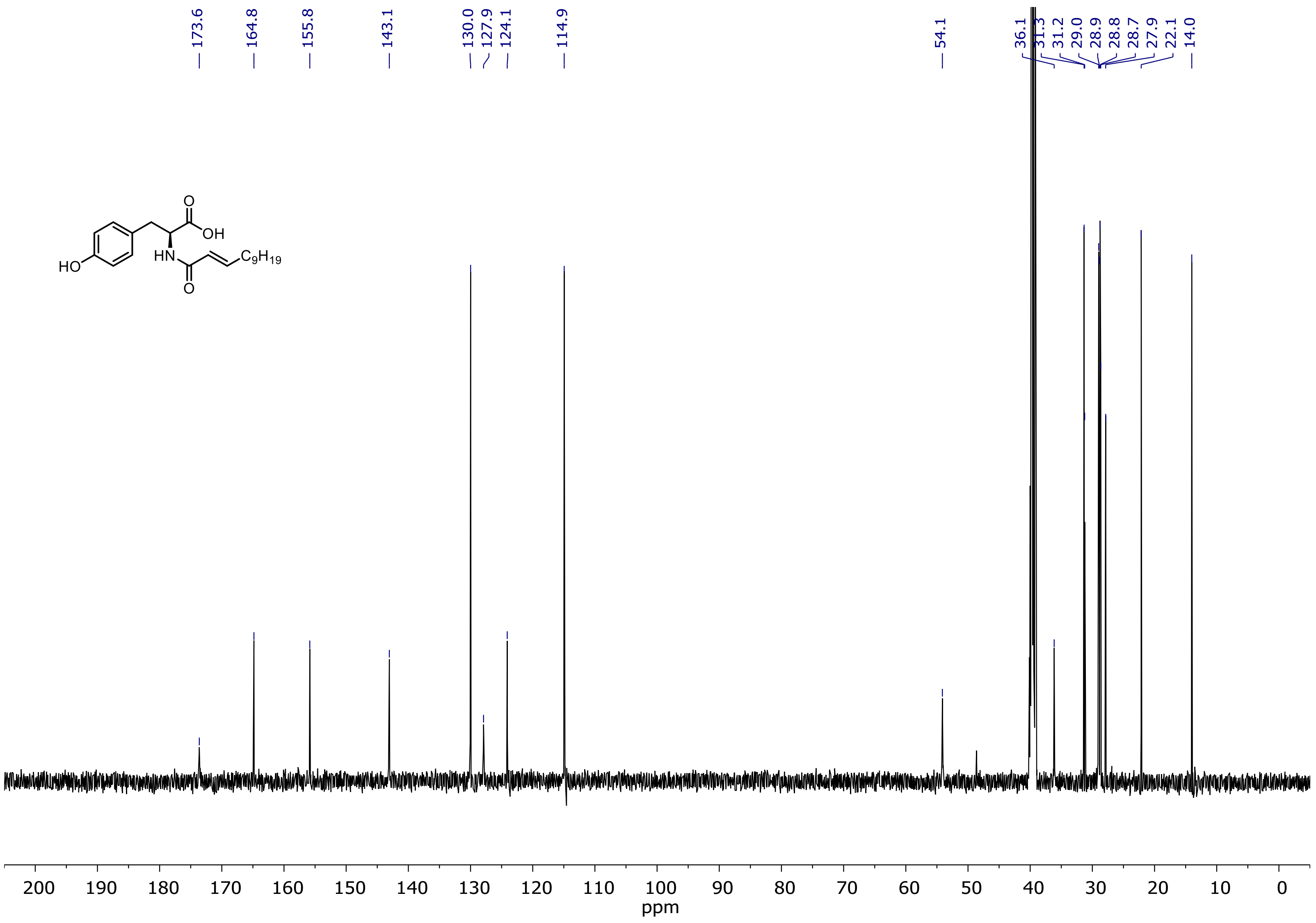

Supplementary Figure 33. $^{13}C\{^1H\}$-NMR (126 MHz, DMSO-d6) of (*E*)-dodec-2-enoyl-*L*-tyrosine.

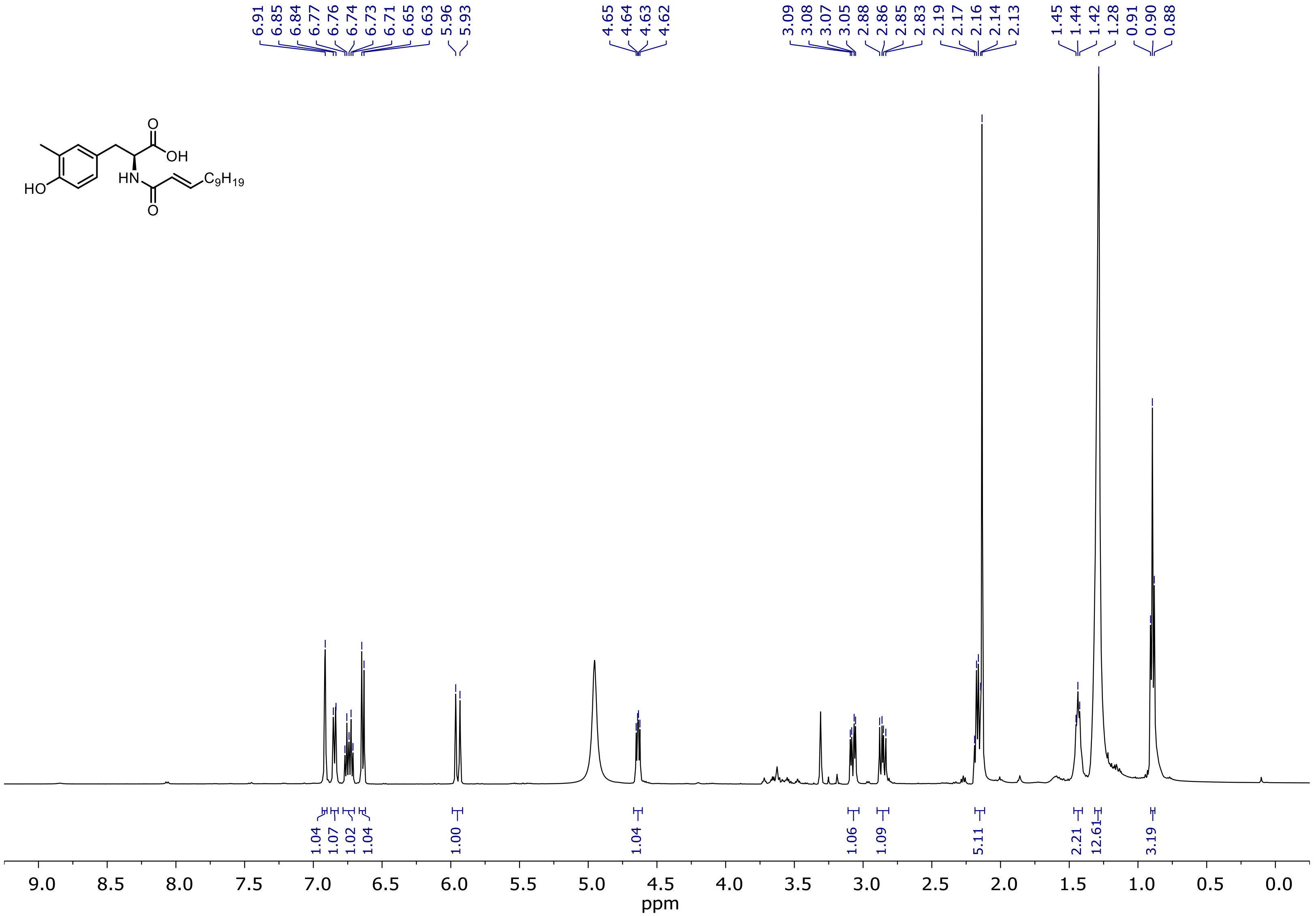

Supplementary Figure 34. ^{1}H-NMR (500 MHz, CD_3OD) of (*S*,*E*)-2-(dodec-2-enamido)-3-(4-hydroxy-3-methylphenyl)propanoic acid.

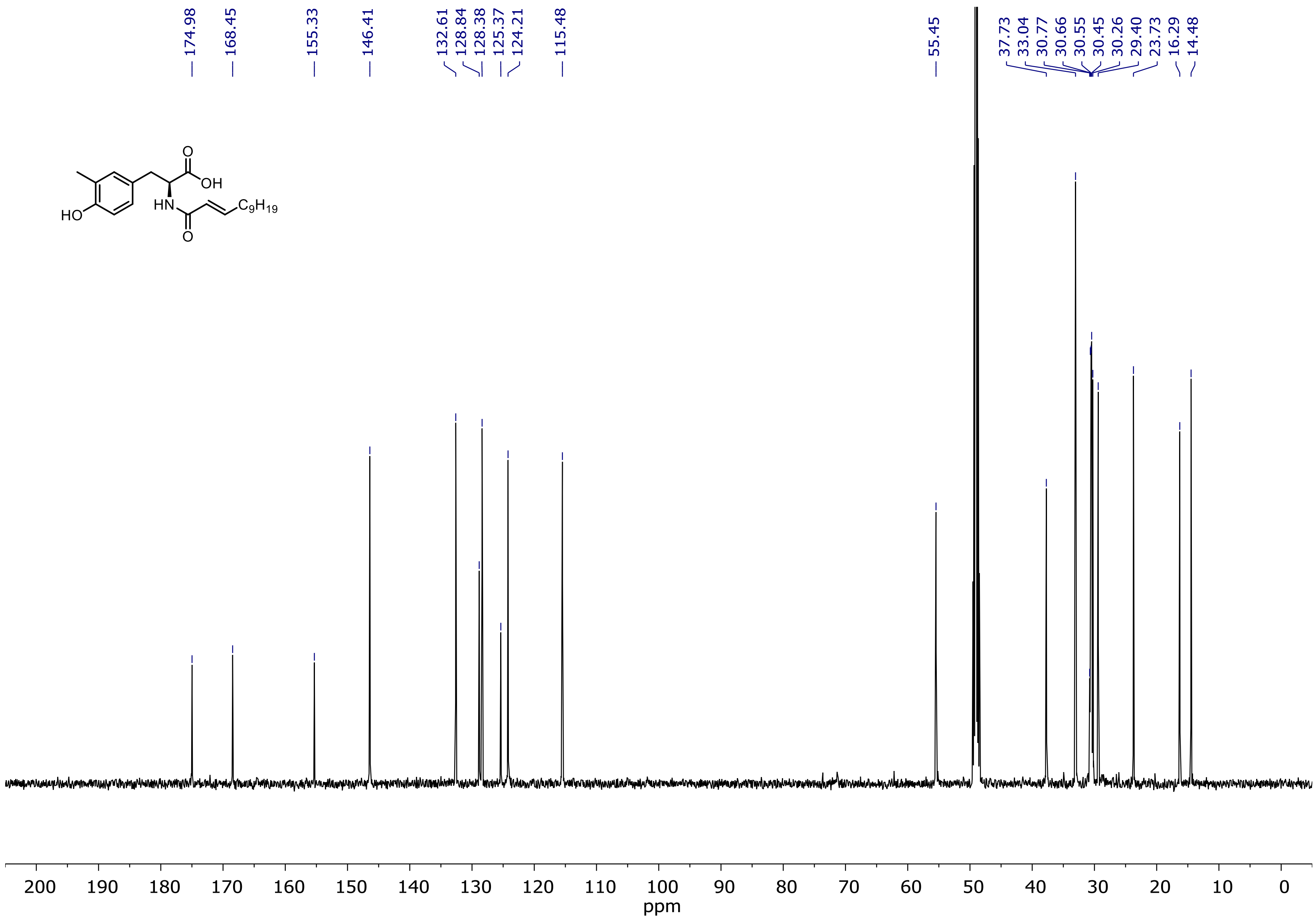

Supplementary Figure 35. $^{13}C\{^{1}H\}$-NMR (126 MHz, CD_3OD) of (*S*,*E*)-2-(dodec-2-enamido)-3-(4-hydroxy-3-methylphenyl)propanoic acid.

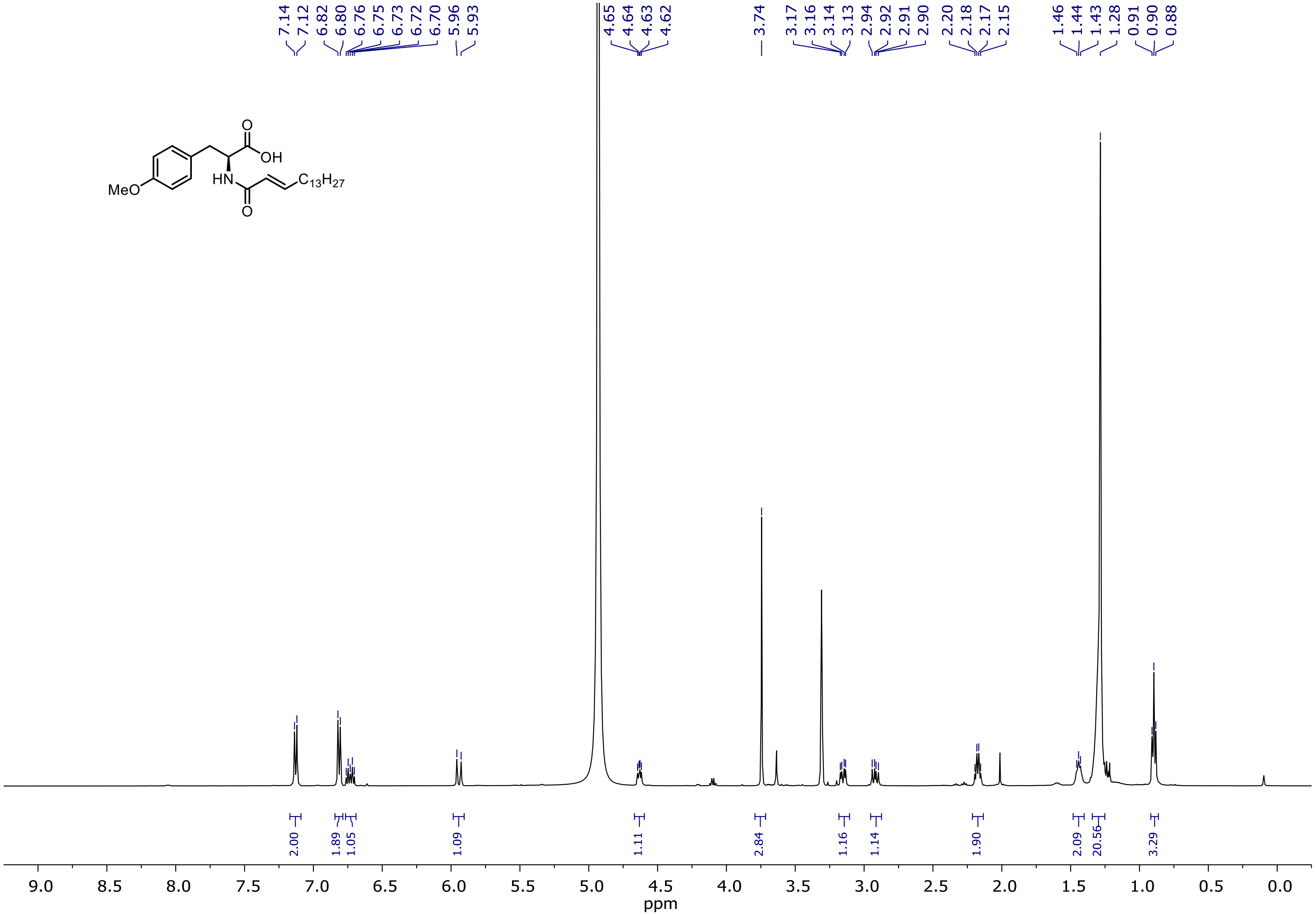

Supplementary Figure 36. ^{1}H-NMR (500 MHz, CD_3OD) of (*S*,*E*)-2-(hexadec-2-enamido)-3-(4-methoxyphenyl)propanoic acid**.**

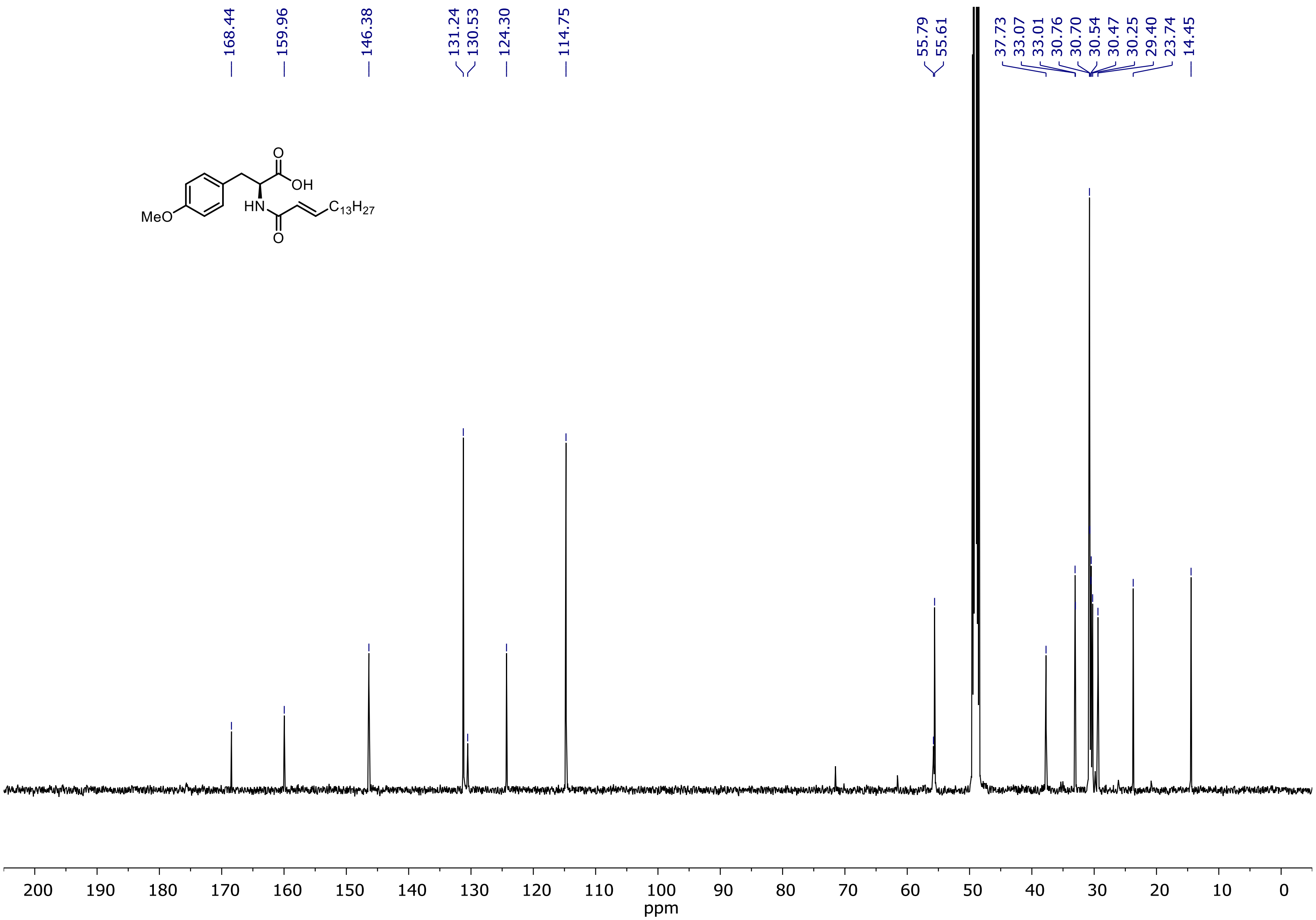

Supplementary Figure 37. $^{13}C\{^{1}H\}$-NMR (126 MHz, CD_3OD) of (*S*,*E*)-2-(hexadec-2-enamido)-3-(4-methoxyphenyl)propanoic acid**.**

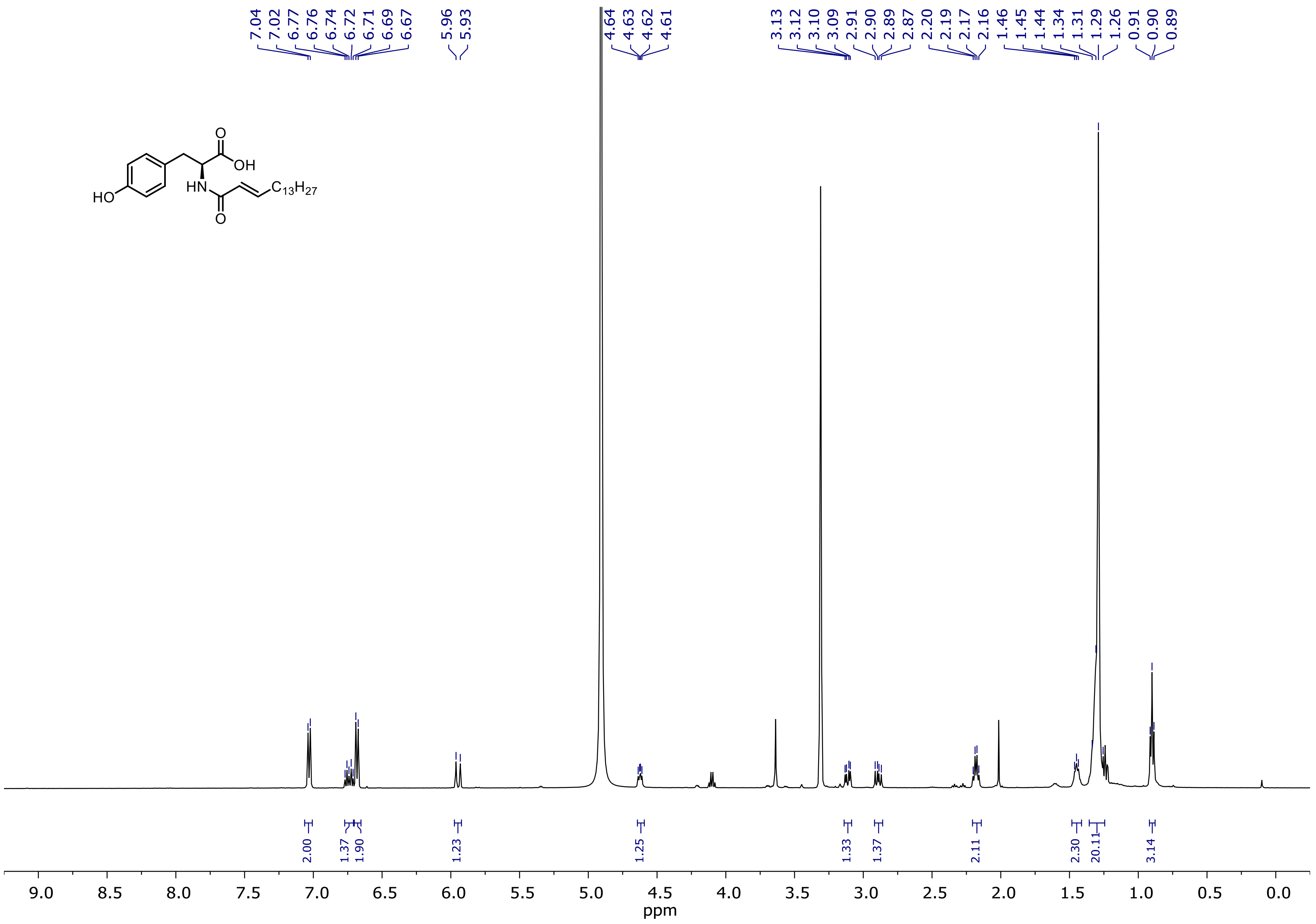

Supplementary Figure 38. ^{1}H-NMR (500 MHz, CD_3OD) of (*E*)-hexadec-2-enoyl-*L*-tyrosine**.**

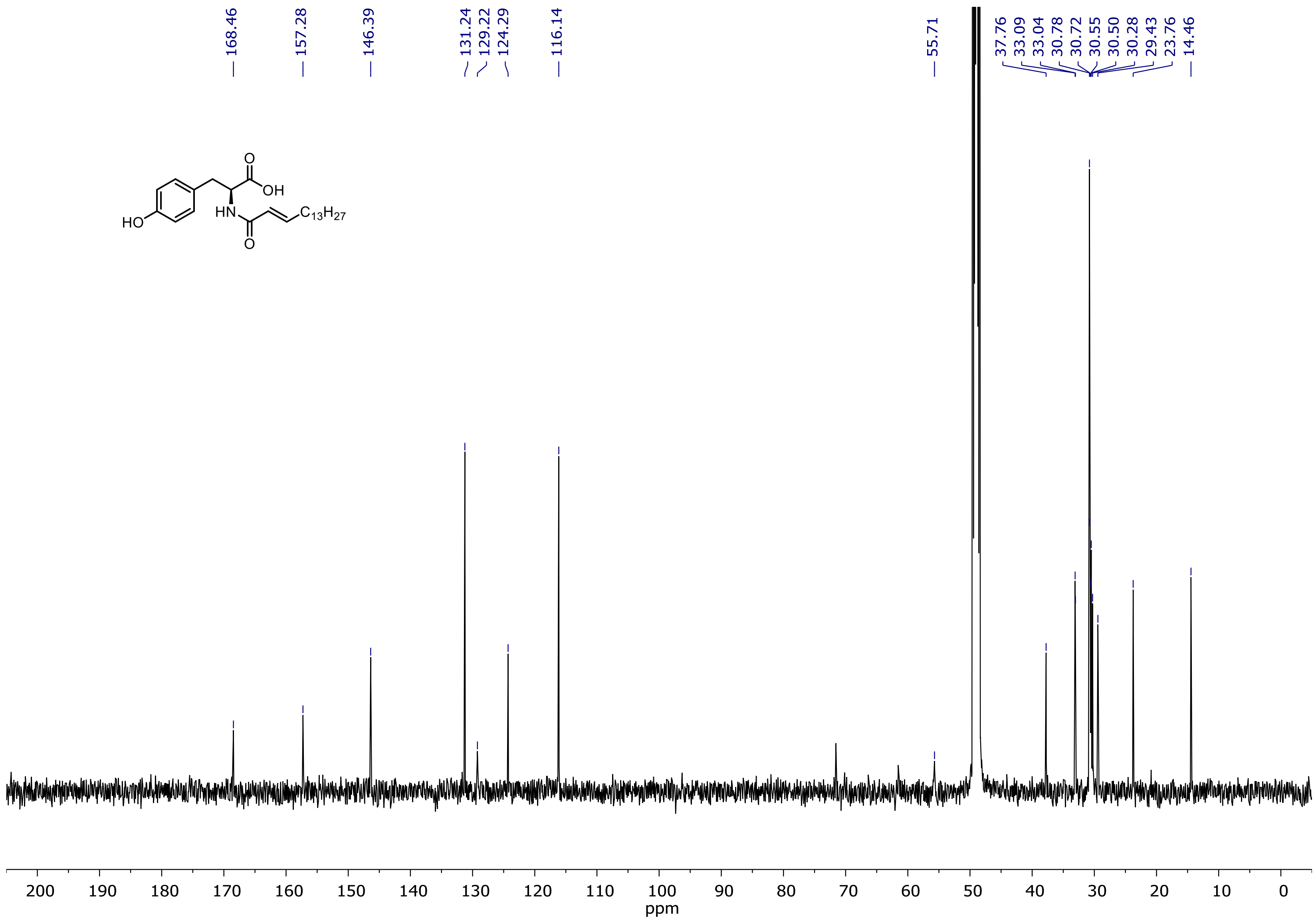

Supplementary Figure 39. $^{13}C\{^{1}H\}$-NMR (126 MHz, CD_3OD) of (*E*)-hexadec-2-enoyl-*L*-tyrosine**.**

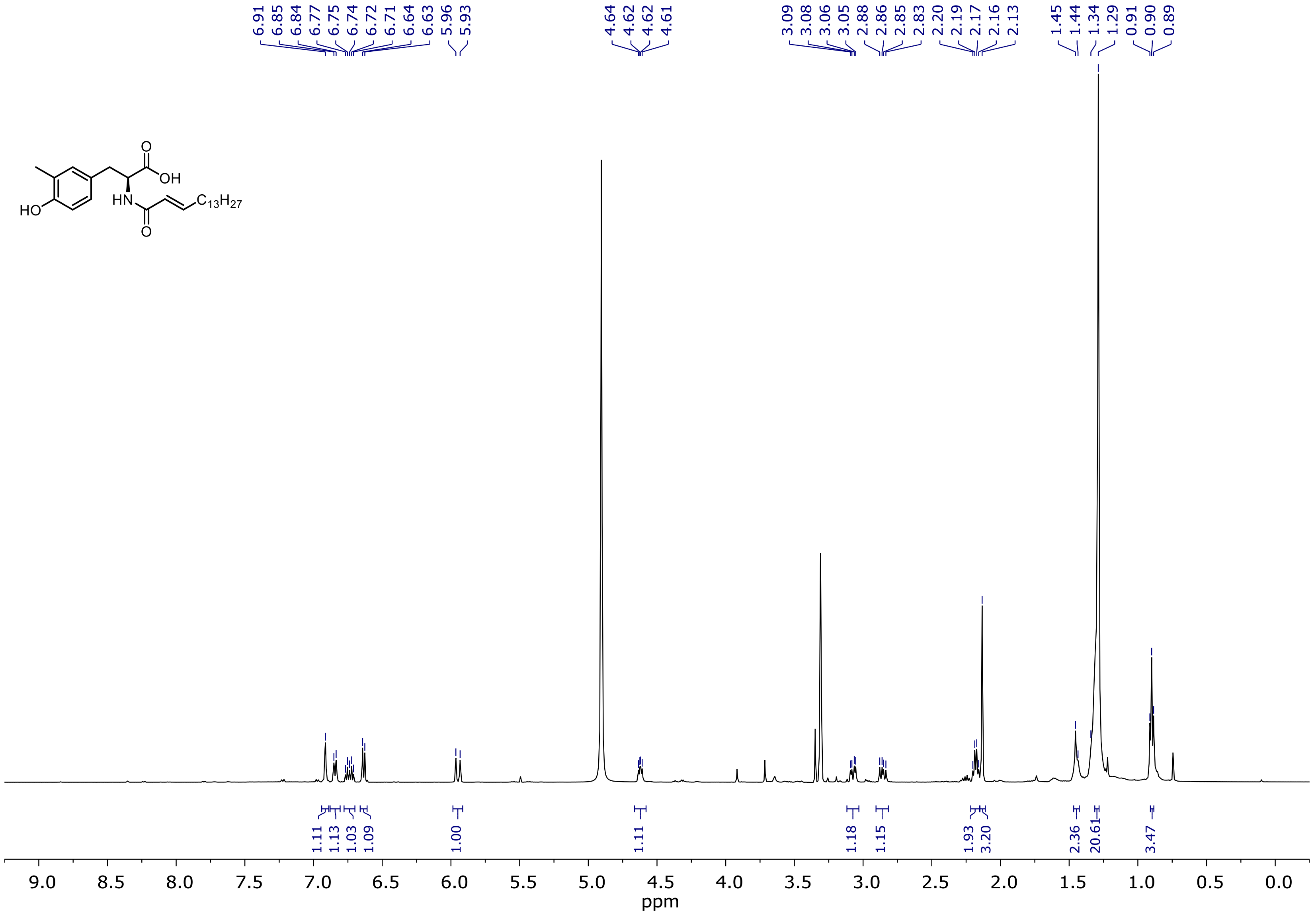

Supplementary Figure 40. ^{1}H-NMR (500 MHz, CD_3OD) of (*S*,*E*)-2-(hexadec-2-enamido)-3-(4-hydroxy-3-methylphenyl)propanoic acid**.**

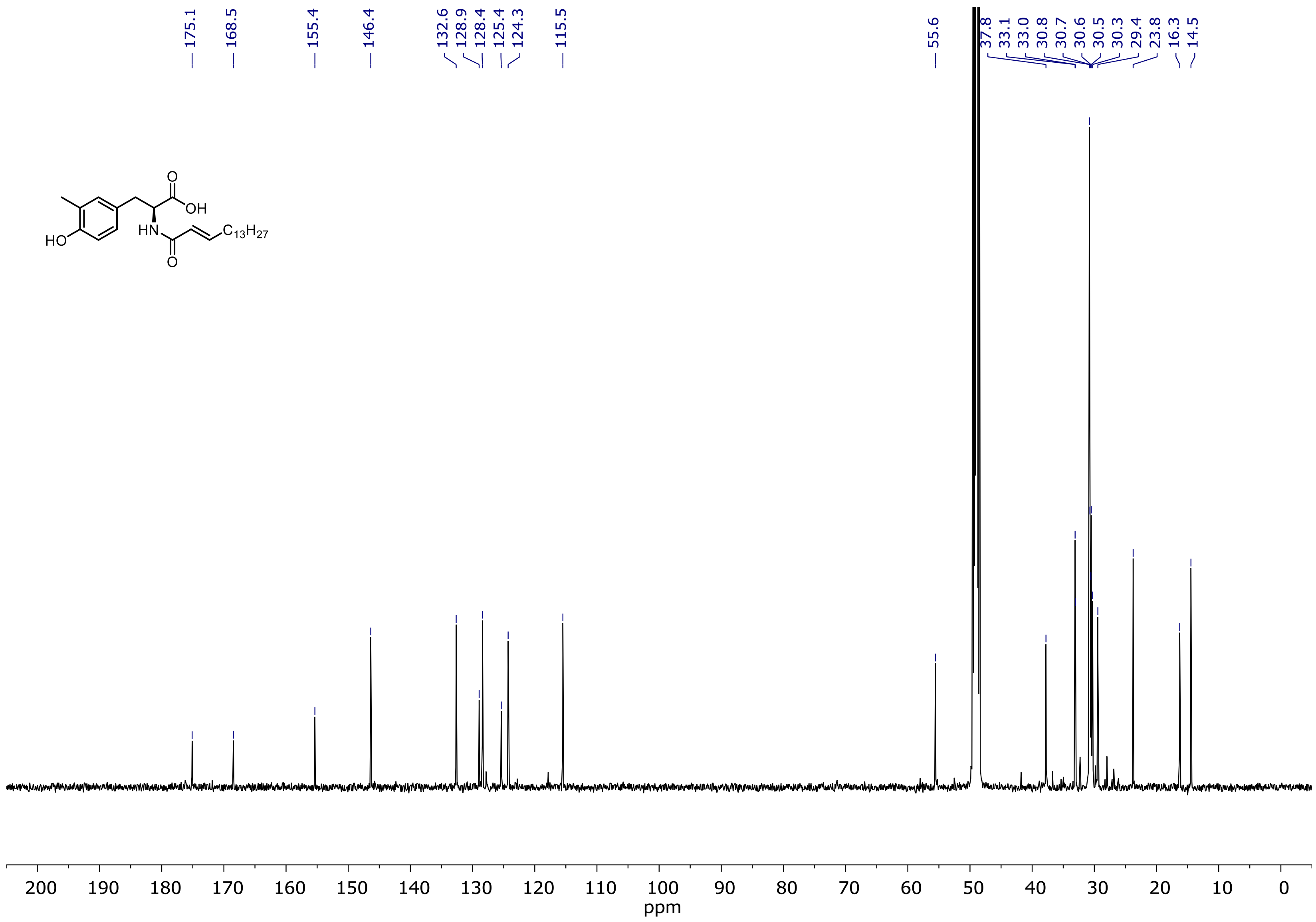

Supplementary Figure 41. $^{13}C\{^{1}H\}$-NMR (126 MHz, CD_3OD) of (*S*,*E*)-2-(hexadec-2-enamido)-3-(4-hydroxy-3-methylphenyl)propanoic acid**.**

Supplementary Table 3. Testing of synthetic compounds for antimicrobial activity. All compounds were dissolved in MeOH and tested at a concentration of 1.0 mg/mL. Ec: *E. coli* SG458, Pa: *P. aeruginosa* K799/61, MRSA: *S. aureus* 134/94 (MRSA), Ef: *E. faecalis* 1528 (VRSA), Mv: *M. vaccae* 10670, Ss: *S. salmonicolor* 549, Ca: *C. albicans* C.A., Pn: *P. notatum* JP36. P: colonies in the inhibition zone, p: single colonies in the inhibition zone. A: no to low inhibition, n.t.: not tested. [c] ciprofloxacin (5 µg/mL in H_2O); [d] amphotericin B (10 µg/mL in DMSO/MeOH); [e] MeOH

**compound**[a]	***E. coli*** [mm]	***Pseud.*** [mm]	***MRSA*** [mm]	***VRSA*** [mm]	***M. vaccae*** [mm]	***C. albicans*** [mm]
37	0	0	0	0/A	13p-P	0
30	0	0	0	0	0	0
18	0	0	0	0	0	0
21	0	0/A	15/24P	18/25p	18p	0/A
20	0	0	0	0	0	0
23	0	13P	11/18P	12/20p, F	19	0
24	0	0	0	0	11p	0
27	0	0	13/20p	16/23p	16p	0/A
26	0	0	0	0	0	0
29	0	12P	10	13/18P	13p	0
19	0	0	0	0	11p-P	0
22	0/A	0/A	11	11/21P	12p	0
25	0	0	0	0	0	0
28	0	0/A	13/20P	12/18p-P, F	18	0
positive control	23/32p	25[c]	0[c]	16F[c]	22p[c]	21[d]
negative control[e]	0	0	0	0	0	0

Supplementary References

[1] S. J. Johansson, T. Johannessen, C. F. Ellefsen, M. S. Ristun, S. Antonsen, T. V. Hansen, Y. Stenstrøm, J. M. Nolsøe, *Synlett* **2019**, *30*, 213-217.
[2] M. Rischer, L. Raguž, H. Guo, F. Keiff, G. Diekert, T. Goris, C. Beemelmanns, *ACS Chem. Biol.* **2018**, *13*, 1990-1995.
[3] H. C. Gallantree-Smith, S. G. Antonsen, C. H. Görbitz, T. V. Hansen, J. M. Nolsøe, Y. H. Stenstrøm, *Org. Biomol. Chem.* **2016**, *14*, 8433-8437.
[4] J. M. Richter, B. W. Whitefield, T. J. Maimone, D. W. Lin, M. P. Castroviejo, P. S. Baran, *J. Amer. Chem. Soc.* **2007**, *129*, 12857-12869.
[5] A. Pal, Y. K. Ghosh, S. Bhattacharya, *Tetrahedron* **2007**, *63*, 7334-7348.
[6] S. Basak, J. Nanda, A. Banerjee, *Chem. Commun.* **2014**, *50*, 2356-2359.
[7] D. Beaufils, G. Danger, L. Boiteau, J.-C. Rossi, R. Pascal, *Chem. Commun.* **2014**, *50*, 3100-3102.
[8] N. Kallscheuer, O. Jeske, B. Sandargo, C. Boedeker, S. Wiegand, P. Bartling, M. Jogler, M. Rohde, J. Petersen, M. H. Medema, *Commun. Biol.* **2020**, *3*, 303.
